# Bionic Multimodal Augmented Somatosensory Receptor Enabled by Thermogalvanic Hydrogel

**DOI:** 10.1002/advs.202505873

**Published:** 2025-06-29

**Authors:** Ning Li, Zhaosu Wang, Yu Niu, Yu Li, Suyi Wen, Hulin Zhang, Zong‐Hong Lin

**Affiliations:** ^1^ College of Integrated Circuits Taiyuan University of Technology Taiyuan 030024 China; ^2^ College of Electronic Information Engineering Taiyuan University of Technology Taiyuan 030024 China; ^3^ Department of Biomedical Engineering National Taiwan University Taipei 10167 Taiwan

**Keywords:** deep learning, fingertip receptor, skin‐inspired, tactile perception, thermogalvanic hydrogel

## Abstract

The emergence of an e‐skin receptor is an optimal solution for restoring the hand function of patients with sensation disorder, while constructing an e‐skin receptor with high sensitivity, self‐supervised capability, and open‐environmental stability remains challenging. Here, inspired by the human skin perception mechanism, an ultrasensitive self‐powered multimodal fingertip receptor that integrates thermogalvanic hydrogels as active mechanoreceptors and thermoreceptors for entropy‐stabilized material fingerprint perception is proposed. A micropatterned and gradient structure strategy is introduced to improve the sensitivity to 53.6 kPa^−1^ with a low detection limit of 1.9 Pa. By exploiting static thermovoltage and dynamic differential signals to visualize the unsteady interfacial heat conduction, different materials can be determined in 80 ms based on the fast and slow adaptive sensations of the receptor. The self‐supervised thermovoltage compensation is realized by self‐decoupling contact pressure and thermal contact coefficients of materials, accommodating variations in applied forces. Benefiting from the robust interfacial heat transfer process and thermoelectric conversion, the tactile perception mechanism demonstrates universality under various external surroundings and contact conditions. With the assistance of deep learning, the fingertip receptor can function as an augmented somatosensory receptor to accurately perceive cutaneous cues of objects with an accuracy of 95.5%, which provides the potential of intelligent haptic perception to human‐machine interfaces and prosthetics.

## Graphical abstract

A skin‐inspired self‐powered multimodal fingertip receptor that integrates thermogalvanic hydrogels as active mechanoreceptors and thermoreceptors is proposed. By exploiting dynamic‐static thermovoltage of the hydrogel to visualize the unsteady interfacial heat conduction, different materials can be determined in 80 ms. The self‐supervised thermovoltage compensation is realized by self‐decoupling contact pressure and thermal contact coefficients of materials, accommodating variations in applied forces.

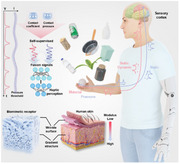

## 1 Introduction

Skin serves as the essential interface and protective barrier between the human body and surrounding environment.^[^ ^1^, ^2^, ^3^ ^]^ The combination of sensory perception and mechanical deformability of skin facilitates seamless detection and response to external stimuli, enabling the execution of intricate tasks in both dynamic and unpredictable world.^[^ ^4^, ^5^, ^6^ ^]^ Unfortunately, patients experiencing skin damage or peripheral neuropathies often face significant sensory disorders, which significantly hampers basic interactions with objects, such as recognizing materials, contact pressures, and locations.^[^ ^7^, ^8^, ^9^ ^]^ Prosthetic limbs, while offering some restoration of sensation functions, are hindered by issues including phantom limb pain, bulky size, and limited dexterity, which needs to be addressed through the incorporation of lightweight materials, concise structures and tissue compliance.^[^ ^10^, ^11^ ^]^ To enable a natural e‐skin receptor, efforts have been made to confer (e.g., tactile sensation, multimodal signal decoupling, system design and integration) on electronic systems with skin‐like performance (e.g., soft and deformable).^[^ ^12^, ^13^, ^14^, ^15^, ^16^ ^]^

Existing research on tactile sensation using smart fingers and wearable tactile gloves incorporating triboelectric, capacitive, piezoresistive, and piezoelectric sensors has shown possibility for aiding interactions during recovery from sensation disorders.^[^ ^17^, ^18^, ^19^, ^20^ ^]^ Piezoresistive and piezoelectric sensors have gained wide interests for their simple structure and easy fabrication. These characteristics make them ideal candidates for crafting pressure sensors, capable of swiftly generating electrical signal variations in response to mechanical stimuli.^[^ ^21^, ^22^ ^]^ However, these piezoresistive and piezoelectric sensors fail to recognize materials and achieve self‐supervised tactile perception in the absence of a preset net force or displacement. In parallel, a few artificial tactile sensors based on triboelectric and capacitive effects have been reported to perceive attributes related to material and pressure.^[^ ^23^, ^24^, ^25^ ^]^ Although the sensors can limitedly distinguish objects of different sizes or materials, they struggle with identifying objects with similar electronegativity or dielectric constants and their output performance is susceptible to the ambient environment.^[^ ^26^ ^]^ Additionally, the piezoresistive and capacitive sensors require external power sources for sensation, increasing the complexity and potential bulk of wearable bioelectronics. Therefore, it is urgent to develop self‐powered multifunctional flexible tactile sensors with self‐supervised capability and environmental stability to improve the accuracy of tactile perception.

In this research, inspired by the tactile perception mechanism of the skin, we present a wearable and self‐powered multimodal fingertip receptor based on thermogalvanic hydrogels as a fundamentally new platform technology for an augmented somatosensory receptor. The fingertip receptor allows patients with sensation disorder and robotic arms to perceive material and pressure through thermogalvanic and active piezoresistive effects, respectively. We created a gradient and micropatterned structure on the thermogalvanic hydrogel (GMTH) with enlarged vertical compress deformation, which exhibits high pressure sensitivity (53.6 kPa^−1^), a short response time (110 ms) and wide sensing range (0–500 kPa). Meanwhile, a high toughness of 26.22 MJ m^−3^ and strain of 611.4% enhance the reliability and lifetime under various deformations in practical applications. The material recognition mechanism of the fingertip receptor is derived from thermovoltage variations induced by the unsteady interfacial heat conduction when contacting different materials. The static thermovoltage and dynamic differential signal based on the hydrogel's thermoelectricity enable visualization of thermal contact coefficients for different materials in 80 ms. The self‐supervised strategy of decoupling the contact pressure and thermovoltage is utilized to compensate the thermovoltage under various applied forces. The tactile perception mechanism works stably under various external surroundings and contact conditions, enabling entropy‐stabilized material recognition in an open environment. As a proof of concept, we have demonstrated that the intelligent fingertip receptor is able to perform self‐supervised tactile perception with an accuracy of 95.5%. This study provides a potential way of facilitating tactile rehabilitation in patients with dysfunctional skin sensation during recovery, potentially improving their overall quality of life.

## 2 Results and Discussion

### 2.1 Design of the Bionic Multimodal Fingertip Receptor

**Figure 1:**
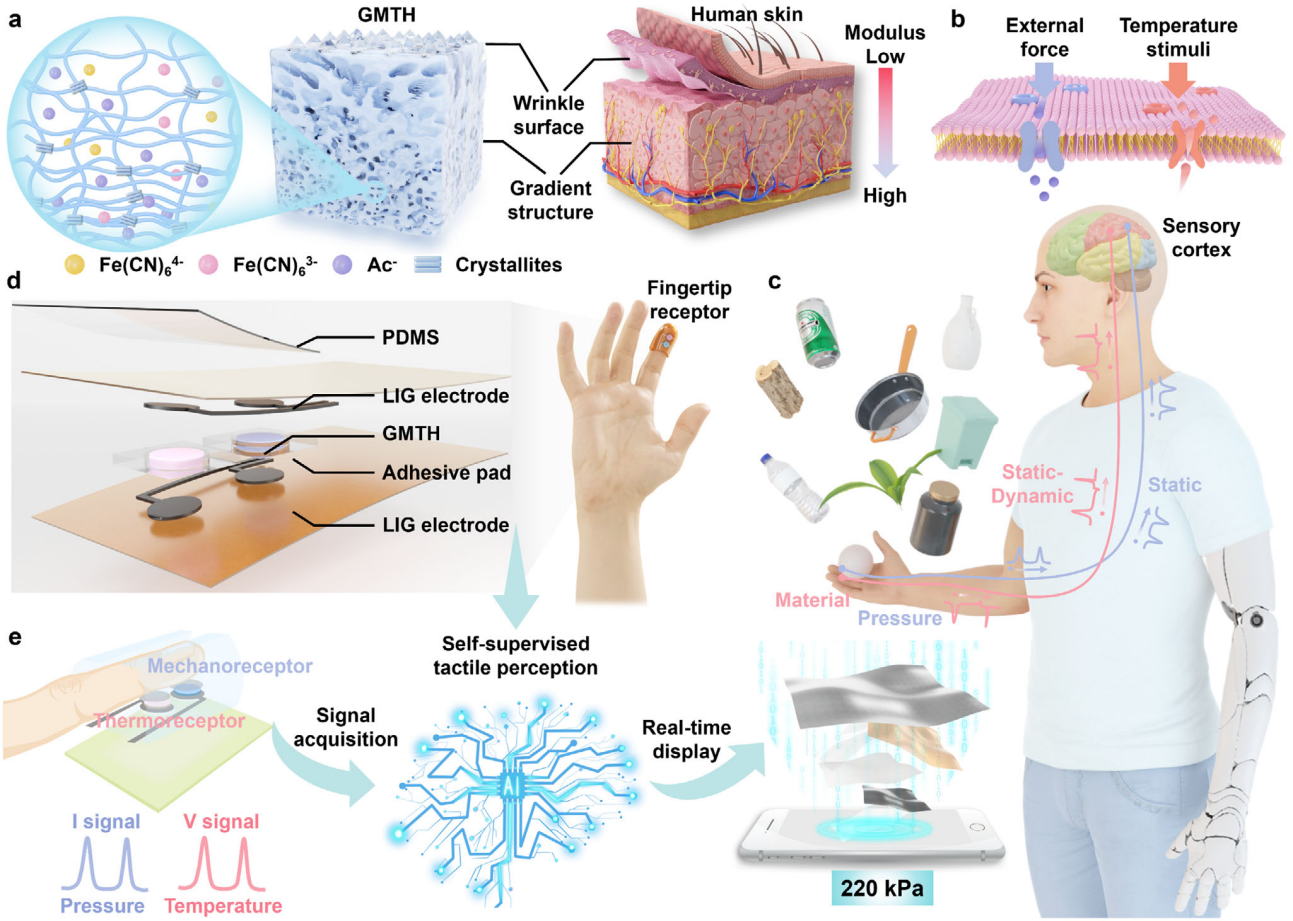
Design of the bionic multimodal fingertip receptor. a) Schematic illustration depicting the human skin‐inspired thermogalvanic hydrogels with micro‐pyramid and gradient structures. b,c) The temperature and pressure perception mechanisms of the human skin. d) The detailed configuration of the proposed fingertip receptor. e) The overview of self‐supervised multimodal tactile perception with the assistance of a deep learning algorithm.

Sensitive modules and structural designs are essential for the multifunctionality and performance of flexible receptors.^[^ ^27^, ^28^ ^]^ Inspired by human skins, a micropatterned thermogalvanic hydrogel with gradient modulus has been fabricated by coupling the template method with directional salting‐out strategy (**Figure** **1**a). The hydrogel after a freezing‐thawing process was placed on the top surface of KAc solution at 253 K f for 12 h to achieve directional ion diffusion (Figure S1a, Supporting Information). Due to the bottom‐up directional diffusion, the K^+^ and Ac^−^ ions form a concentration gradient to induce the PVA chains agglomerating in the vertical direction. The cross‐section scanning electron microscopy (SEM) images in Figure S1b (Supporting Information) can prove the pore‐gradient structure of the hydrogel. By contrast, the hydrogel fully immersed in KAc solution presents a homogeneous porous structure (Figure S1c, Supporting Information).

The thermoreceptors and mechanoreceptors in the finger skin are capable of sensing temperature and pressure stimuli when touching different objects (Figure 1b). Subsequently, fast and slow adaptive receptors generate sensory signals regarding tactile information, which are further encoded into spike sequences (static or dynamic) and sent to the sensory cortex cerebral nervous system for accurately recognizing objects (Figure 1c). Inspired by the multimodal tactile perception of the finger skin, a wearable multimodal fingertip receptor utilizing the synergy of thermogalvanic and active piezoresistive effect was designed for self‐supervised material perception in an open environment. Figure 1d depicts the structure of the receptor with four layers. The top layer serves as the thermal insulator for eliminating the influence of environmental temperature variations on pressure sensing. The sensing layer, located at the middle part of the receptor, decouples the pressure and temperature difference into two distinct electrical signals (current and voltage variations). Two GMTHs are integrated into sensitive modules for mimicking the thermoreceptors and mechanoreceptors. The other layers are laser‐induced graphene (LIG) electrodes with superior flexibility, electrical conductivity (≈4000 S m^−1^), and hydrophilicity (Figure S2, Supporting Information). The fingertip receptor is compact, with a thickness of ≈1 mm and a weight of ≈0.523 g. Additionally, the fully conformal configuration ensures the comfortability of the fingertip receptor while maintaining its functionality and user‐friendliness. When a finger equipped with the receptor contacts an object, the receptor can generate dynamic‐static electrical signals corresponding to material and pressure, which are collected in real‐time and transmitted for deep‐learning‐assisted data analysis to fulfill a highly accurate tactile perception (Figure 1e). The thermovoltage can be compensated by self‐decoupling contact pressure and thermovoltage when identifying the material types. Consequently, the material perception of the fingertip receptor is independent of applied external force under the integration of pressure module, exhibiting the self‐supervised ability for entropy‐stabilized tactile perception.

### 2.2 Mechanical Performance Characterization of the GMTH

**Figure 2:**
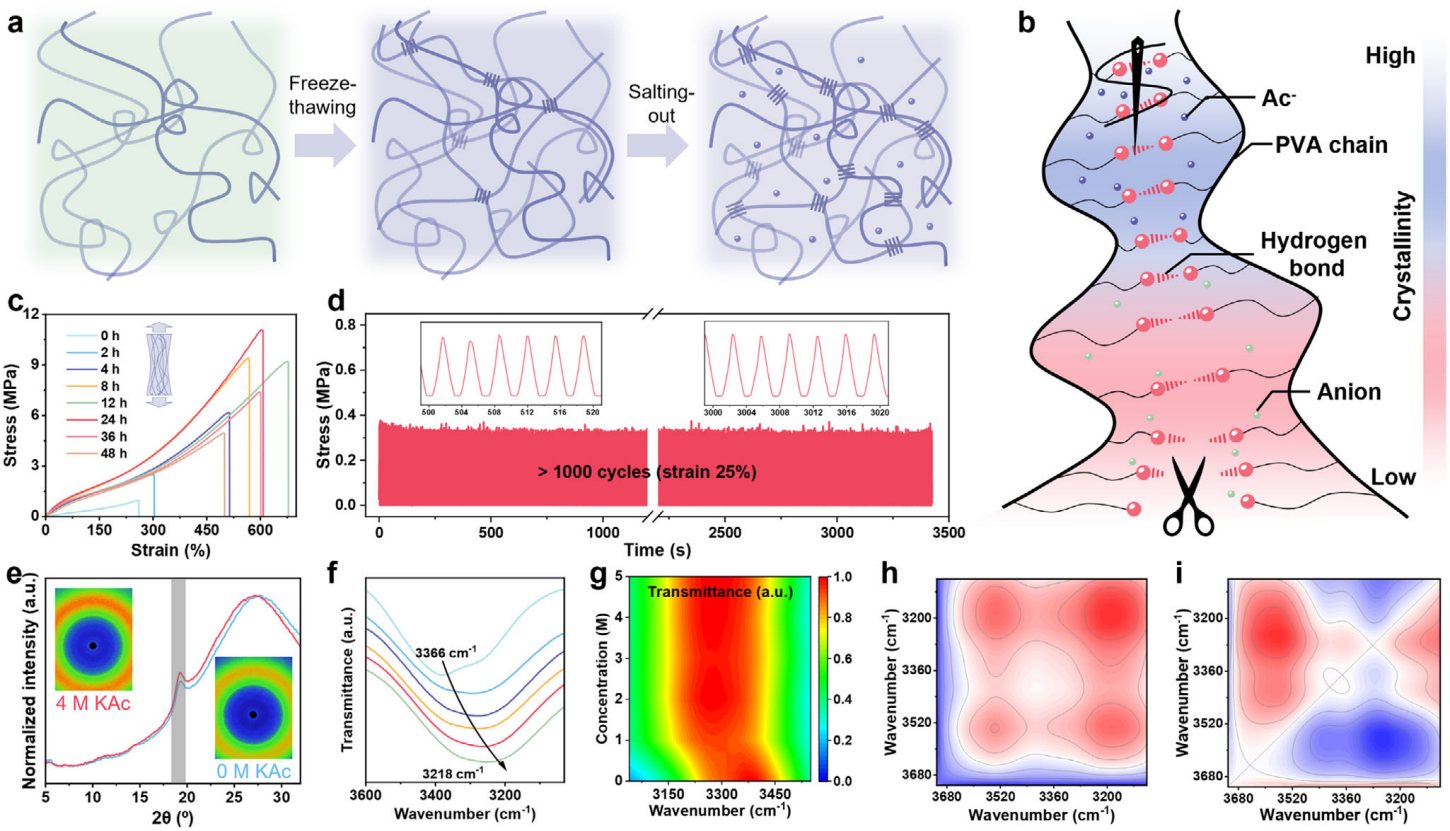
Mechanical performance characterization of the GMTH. a) Fabrication procedure of the GMTH. b) The mechanism of regulating mechanical property based on the salting‐out strategy. c) The stress‐strain profiles with different immersion times at 4 m KAc solution. d) Robustness tests of the GMTH under the successive compression of 25% for over 1000 cycles. e) 2D WAXS patterns and 1D WAXS profiles of hydrogels with and without KAc. f,g) The red‐shifted O─H stretching vibration peak of the hydrogel with various KAc concentrations from 3366 to 3218 cm^−1^. h,i) 2DCOS synchronous and asynchronous spectra generated from temperature‐variable FTIR. Red and blue areas represent positive and negative values, respectively, with intensity increasing as the color becomes more vivid.

Poly(vinyl alcohol) (PVA) is chosen as the raw material because it can form a high density of intermolecular hydrogen bonds.^[^ ^29^, ^30^ ^]^ We synergistically employ the freeze‐thawing process and salting‐out effect to produce a mechanically robust and environmentally stable thermogalvanic hydrogel (**Figure** **2**a). During the freeze‐thawing process, the PVA is fixed in a specific shape macroscopically and the polymer chains are prepacked microscopically to facilitate the subsequent aggregation induced by ions. Salting‐out ions, such as potassium ion (K^+^) and acetate (Ac^−^), are effective in promoting the intensive aggregation and crystallization of PVA chains through the Hofmeister effect to form a hydrogel with higher crystallinity (Figure 2b). This aligns with the order in water content of hydrogels after soaking in different KAc concentrations (Figure S3, Supporting Information).

The mechanical property of the GMTH can be readily tuned by tailoring KAc concentration and immersion time, enabling precise control over the aggregation and crystallization of polymer chains. After soaking 12 h in 4 KAc solution, the hydrogel boasts a remarkable weight‐bearing capacity exceeding 3500 times its own capacity (Figure S4a, Supporting Information). The well‐designed crystalline domain and chain alignment enable the GMTH to withstand various types of deformation, including twisting, knotting and cross stretching without breaking (Figure S4b, Supporting Information). Further regulating the salt concentration and salting‐out time contributes to simultaneous improvements in toughness (1.7–29.1 MJ m^−3^), tensile strength (0.95–11.8 MPa), high cyclic stability (>500 cycles), and elongation at break (259.9–676.7%), as depicted in Figures 2c and S5 (Supporting Information). The numerous crystalline domains, acting as rigid cross‐linkers, effectively delay the fracture of polymer chains by crack blunting and pinning effects.^[^ ^31^ ^]^ The fracture energy ranges from 130.6 to 2510.7 kJ m^−2^ as the KAc concentration increases from 0 to 5 m, which is in accordance with the above results of mechanical tests (Figure S6, Supporting Information). Energy dissipation is examined under successive cyclic loading without intervals between cycles (Figure S7a, Supporting Information). As tensile strain increases, the dissipation energy of the GMTH sharply rises from 0.16 MJ m^−3^ at 50% strain to 2.8 MJ m^−3^ at 350% strain, revealing that a great deal of energy is dissipated by the fracture of hydrogen bonds (Figure S7b, Supporting Information). In addition, the loading curve crosses the last unloading curve during continuous loading‐unloading, indicating obvious self‐recovery thanks to partially reconstructed hydrogen bonds.

To verify the formation of hydrogels with gradient modulus, the compression performance has been systematically investigated. The shapes of the GMTH soaked in different KAc concentrations at 253 K were recorded in Figure S8a (Supporting Information), showing the KAc concentration has a prominent effect on the gradient modulus of the GMTH. From the J‐shaped stress‐strain curves in Figure S8b–d (Supporting Information), the compressive modulus increases with the rise of ion concentration, soaking time, and diffusion temperature, which is mainly ascribed to the content of diffused ions in the hydrogel. Fingertip receptors suitable for tactile perception should maintain outstanding fatigue resistance and fast recovery. As illustrated in Figure 2d and Figure S8e (Supporting Information), the GMTH can endure a large cyclic strain of 50% and recover from 1000 consecutive compression cycles with 25% strain, indicating that the remarkable elasticity of the hydrogel and lying a solid foundation for pressure sensing. The mechanical improvement after immersing in the KAc solution is further confirmed by the wide‐angle X‐ray scattering (WAXS) and Fourier transform infrared (FTIR) spectra. The peak intensity in WAXS profiles is enhanced, indicating improved crystallinity after immersion in 4 m KAc solution (Figure 2e). Notably, the average size of crystalline domains remains unchanged, which suggests that the increased crystallinity is primarily attributed to the prompting quantity of crystalline domains instead of individual domain size. Owing to the small volume of PVA crystallites induced by the freeze‐thawing and salting‐out strategy, the GMTH retains high transparency with little visible light being scattered (Figure S9, Supporting Information). As demonstrated in Figure 2h,i, the redshifts of the characteristic peaks of the PVA hydroxyl groups indicate the intermolecular hydrogen bonds are enhanced, promoting the emergence of crystallization of polymer chains. Moreover, the portion of crystalline domains is directly reflected by the absorption peaks of 1143 cm^−1^ and the hydrogels soaked in KAc solution exhibit the higher peak intensity, which are the fingerprint of PVA crystallinity (Figure S10a, Supporting Information). To further elucidate the structural role of hydrogen bonding in the hydrogel matrix, we conducted temperature‐variable FTIR combined with generalized 2D correlation spectroscopy (2DCOS). As the temperature increases from 303 K to 353 K, the intensity of the crystalline peak at 1142 cm^−1^ declines, suggesting the melting of PVA crystalline domains (Figure S10b, Supporting Information). Simultaneously, the absorption intensity of the hydroxyl groups stretching band at 3245 cm^−1^ gradually decreases, accompanied by a shift to higher wavenumbers (Figure S10c, Supporting Information). This observation indicates the thermal disruption of hydrogen bonding, consistent with the well‐known thermoresponsive nature of hydrogen bonds. Furthermore, 2DCOS analysis reveals that hydroxyl groups in the 3200–3520 cm^−1^ range respond differently to temperature changes (Figure 2h,i). According to Noda's rule, the spectral changes at ≈3200 cm^−1^ occur earlier than those at higher wavenumbers, indicating that strongly hydrogen‐bonded hydroxyl groups respond first. This demonstrates a sequential dissociation process of hydrogen bonds under heat, indicating the existence of hydrogen bond interactions in polymer networks. In brief, the synergy of the freeze‐thawing process and salting‐out effect not only enhances the toughness, elasticity, and elongation at break of the hydrogel but also ensures its potential in flexible and transparent tactile perception.

### 2.3 Thermoelectric Performance Characterization of the GMTH

**Figure 3:**
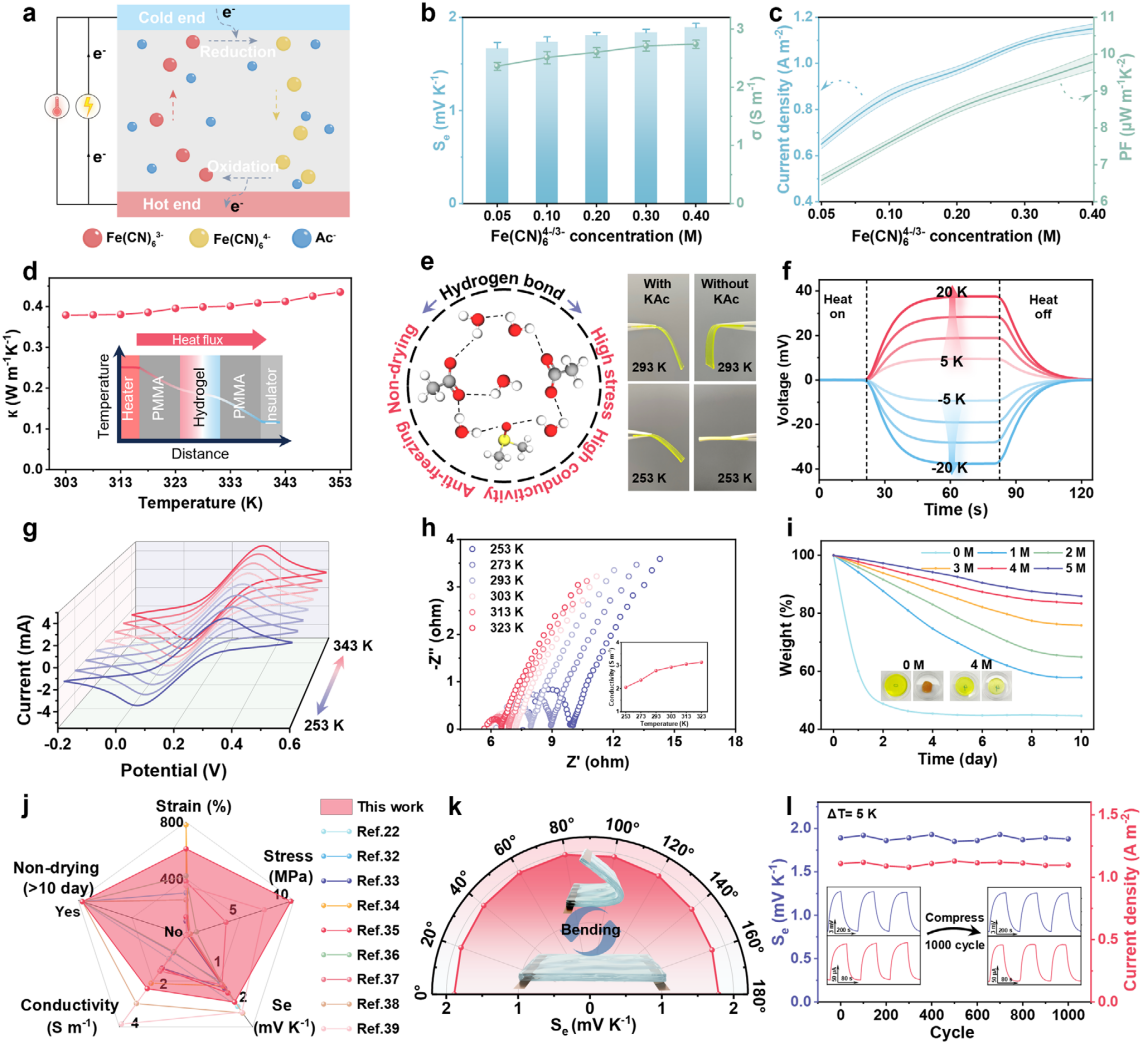
Thermoelectric performance characterization of the GMTH. a) Working mechanism of the GMTH located at the temperature difference. b,c) The effect of [Fe(CN)_6_]^4−/3−^ concentrations on the thermoelectric performance, including S_e_, σ, current density, and PF. d) The fluctuation of the thermal conductivity at different temperatures. e) Hydrogen bonds formed among water molecules, Ac^−^ ions, and residual DMSO endowing the hydrogels with versatility. f) Stability of the thermovoltage under different temperature gradients at 253 K. g) The stability of the redox reactions under various temperatures. h) Dependence of the electrical conductivity on the different temperatures. i) Weight change curves of the hydrogels with different KAc concentrations. j) Comparison of the mechanical and electrical properties between the prepared GMTH and reported thermogalvanic hydrogels. k) Reliability of the thermopower across various bending angles. l) Cyclic compressing test of the GMTH over 1000 cycles at temperature gradient of 5 K. Inset shows the voltage and current signals before and after 1000 cycles.

An experimental setup was developed to evaluate the thermoelectric performance of the GMTH, which was sandwiched between two LIG electrodes with high specific surface area and hydrophilicity (Figure S11, Supporting Information). When a temperature difference exists across the GMTH, the reversible reactions of the redox couples occur on both sides, resulting in a potential difference between the two electrodes (**Figure** **3**a). The highly linear relationship between the square‐rooted scan rate and peak current density of cyclic voltammetry (CV) in Figure S12a,b (Supporting Information) implies that the redox reactions are limited by ionic diffusion. Consequently, we modulated the thermoelectric performance of the hydrogel by changing the concentration of [Fe(CN)_6_]^4−/3−^. With [Fe(CN)_6_]^4−/3−^ concentration increasing, both the thermopower (Seebeck coefficient, S_e_) and electrical conductivity reach peak values of 1.89 mV K^−1^ and 2.74 S m^−1^ at 0.4 m [Fe(CN)_6_]^4−/3−^ (Figure 3b; Figure S12c, Supporting Information). Similarly, the current density and power factor (PF) follow this trend when the temperature difference (ΔT) is 5 K (Figure 3c). Based on these results, we selected 0.4 M [Fe(CN)_6_]^4−/3−^ and 4 m KAc as the optimal concentration for further characterization of the GMTH. The thermoelectric performance of the TGH under different stretching conditions was characterized. As the tensile strain increases, both the current and power density decrease from 1.15 A m^−2^ and 2.7 mW m^−2^–0.55 A m^−2^ and 1.4 mW m^−2^, respectively (Figure S12d, Supporting Information). By controlling the temperature of interelectrode at 293 K respectively, the TGH still maintains a maximum output power density of 62.5 mW m^−2^ when the temperature of cold end is lower than 253 K (Figure S12e,f, Supporting Information). Figure 3d shows the trend of thermal conductivity (κ) of the GMTH measured by the stead‐state method (Note S1, Supporting Information) and it is relatively stable under different temperature states, which is beneficial to create a temperature gradient for thermal sensation.

Due to the formation of multiple intermolecular hydrogen bonds among PVA chains, Ac^−^ ions, residual DMSO, and water molecules, the GMTH is endowed with anti‐freezing and non‐drying performance, high conductivity, and remarkable toughness (Figure 3e). The hydrogel with KAc remains its flexibility but a control sample without KAc is completely frozen at 253 K. As shown in Figure 3f, the GMTH still functions properly at 253 K under various temperature gradients. To further evaluate the temperature tolerance of the GMTH, the conductivity and CV tests are conducted at subzero temperature. The residual conductivity (2.07 S m^−1^) and almost symmetrical CV curves at 253 K demonstrate its significant anti‐freezing capability (Figure 3g,h; Figure S13a, Supporting Information). The weight change curves of the GMTH fabricated with different KAc concentrations are measured to study their water retention ability. The weight of the hydrogel without KAc drastically drops to 48.8% after being stored for 2 days at 293 K. By contrast, as the KAc concentration increases from 1 to 5 m, the leftover weights of the hydrogels increase from 57.9% to 85.9%, which originates from the strong hydrogen bonds among Ac^−^ ions, DMSO, and water molecules (Figure 3i; Figure S13b, Supporting Information). The degradation peak of the GMTH at 100–200 °C becomes much lower, confirming the non‐drying performance of the GMTH is significantly improved in the presence of KAc (Figure S13c, Supporting Information). During cyclic testing for over 2 h, the thermovoltag of the hydrogel show no significant change, which demonstrates the hydrogel possesses excellent durability and stability after repetitive heating and cooling (Figure S13d, Supporting Information). Benefiting from the strong hydrogen bonds, the GMTH shows prolonged working stability over 35 days with 70% initial performance (voltage and current density) retained under 5 K temperature gradient (Figure S13e, Supporting Information). Compared to other reported thermogalvanic hydrogels,^[^ ^22^, ^32^, ^33^, ^34^, ^35^, ^36^, ^37^, ^38^, ^39^ ^]^ the GMTH exhibits significant strengths in strain (611%), stress (10.98 MPa), S_e_ (1.89 mV K^−1^), electrical conductivity (2.74 S m^−1^), and non‐drying performance (>10 days) (Figure 3j; Table S1, Supporting Information). It is critical for the GMTH to minimize interference from external mechanical deformation on the thermal voltage to achieve high reliability of material perception. As shown in Figure 3k,l, the thermopower exhibits high robustness under different bending angles and cyclic compression.

### 2.4 Converting Pressure Stimuli into Analyzable Electrical Signals

**Figure 4:**
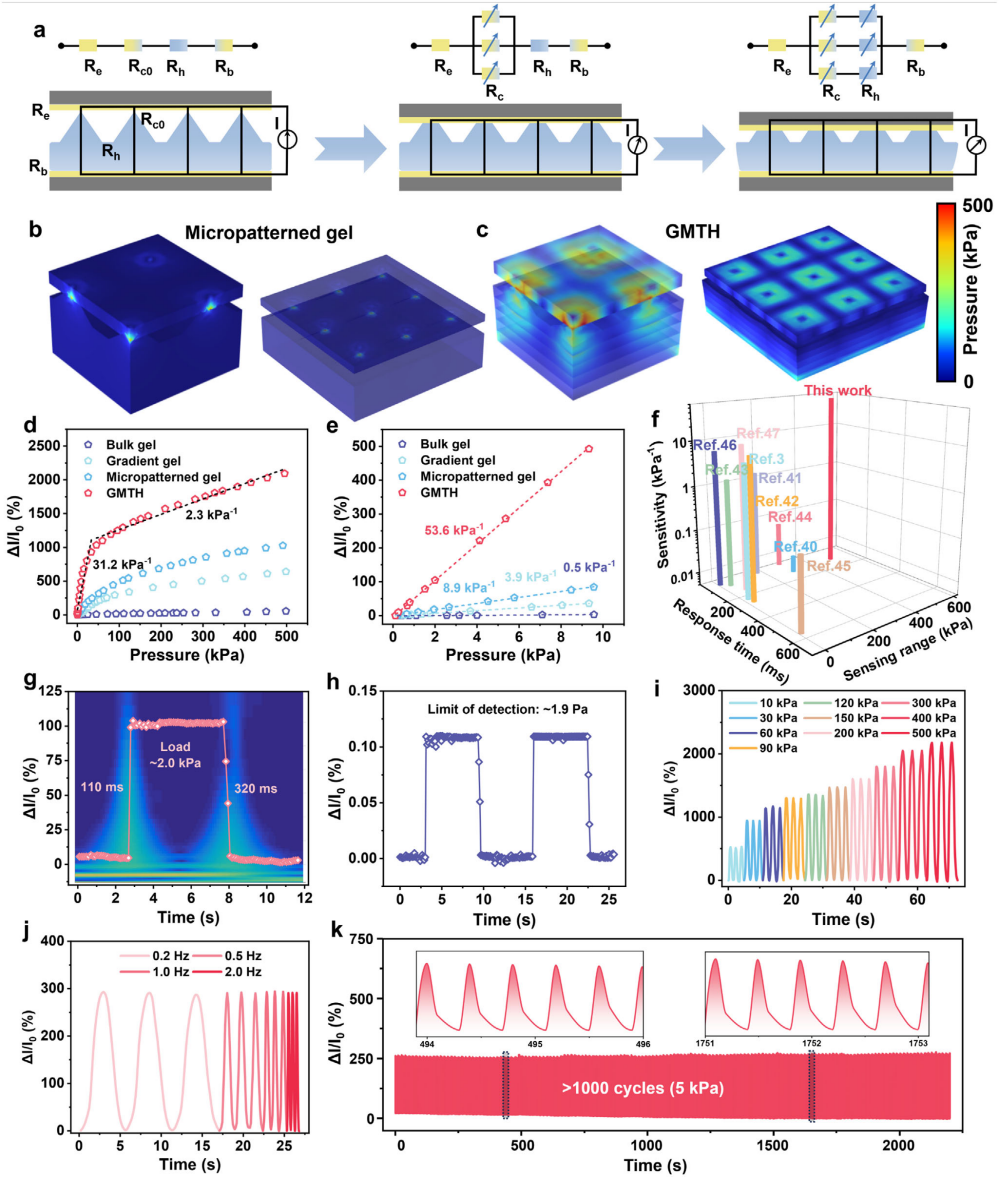
Converting pressure stimuli into analyzable electrical signals. a) Schematic showing the working mechanism of the GMTH. R_e_ is the resistance of electrodes. R_b_ represents the contact resistance between the bottom of the GMTH and the electrode. b,c) Results from COMSOL simulation reveal the stress distribution and maximum deformation. d,e) Pressure‐dependent relative current changes of the hydrogels with different structure configurations and the enlarged view of linear response range between 0 and 10 kPa. f) Sensing performance comparison of the GMTH with previously reported hydrogels in three key parameters. g) Response and recovery times under a pressure of 2 kPa. h) Detection of tiny pressures. i,j) The current output response of the GMTH at various pressures and frequencies. k) Cyclic stability of the sensor with the applied pressure of 5 kPa.

The proposed theoretical model of the active piezoresistive effect is elaborated in Figure S14a,b (Supporting Information). The GMTH functions as a thermocell generating stable thermopower under varying pressures, while its resistance exhibits a change in response to the applied pressure (Figure S14c–e, Supporting Information). This active piezoresistivity enables a transduction from an applied force to a current variation of the thermogalvanic hydrogel under a temperature gradient by the synergistic coupling of the thermogalvanic and piezoresistive effect without external power supplies. While additional power management circuits are essential for processing and filtering the signal, the initial sensing phase is autonomous and does not rely on an external energy supply, thus realizing self‐powered tactile perception. The pressure‐sensitive mechanism of the GMTH has been investigated to elucidate the combination of micro‐pyramid and gradient structure for high sensitivity across a wide range. The pressure‐induced resistance change in the GMTH mainly consists of the variations of the contact resistance (R_c_) at the electrode interface and bulk resistance (R_h_) of the hydrogel. The deformation process can be divided into two stages, as illustrated in **Figure** **4**a. Under low pressure, local stress concentrates at the contact point between the micro‐pyramid and top electrode, causing significant deformation at the tip and a reduction in R_c_ (Figure S14f, Supporting Information). As the applied pressure increases, the lower part of the GMTH undergoes the majority of compressive deformation until there is no cavity between the upper part of the hydrogel and electrode, which produces lower R_c_ and R_h_. To further demonstrate the synergistic design concept, the deformations of the hydrogels without and with gradient modulus upon pressure loading are examined by 3D finite element analysis (Figure 4b,c). The micro‐pyramid structure of the hydrogel contributes to larger resistance variation and higher sensitivity compared with the bulk hydrogel. Under the same pressure, the GMTH with gradient modulus experiences more obvious deformation and possesses the highest sensitivity (Figure 4d). The GMTH achieves a superior sensitivity of 53.6 kPa^−1^ within a linear sensing range of 10 kPa (R^2^ = 0.99) (Figure 4e). As a result, the GMTH with high sensitivity across an ultra‐broad sensing range stands out favorably compared to previously reported studies (Figure 4f; Table S2, Supporting Information).^[^ ^3^, ^40^, ^41^, ^42^, ^43^, ^44^, ^45^, ^46^, ^47^ ^]^

In addition to the high sensitivity over the ultrabroad pressure range, the dynamic response performance of the GMTH has also been thoroughly studied. The response and recovery times under 2 kPa are measured to be 110 and 320 ms, respectively (Figure 4g). Meanwhile, Figure 4h shows that the receptor can reliably detect a tiny pressure of ≈1.9 Pa, which is demonstrated by a distinguishable stepwise increase in current signals. The hysteresis behavior of the pressure sensing performance was evaluated in Figure S15a (Supporting Information). Thanks to the gradient modulus strategy, the GMTH demonstrates a low hysteresis of 16.2% at a pressure of 10 kPa. The rapid response speed, low detection limit, and low hysteresis of the developed GMTH‐based receptor allow it to detect dynamic stimuli, such as artery pulse detection (Figure S15b, Supporting Information). Moreover, the receptor demonstrates stable responses to varying levels and frequencies of dynamic pressure with high repeatability (Figure 4i,j). To assess its reversibility and durability, the response performance of the receptor is evaluated through successive loading and unloading of 5 kPa pressure (Figure 4k). The signal shows minimal changes after 1000 cycles, i.e., the valley and peak current variations increase by 5.26% and 7.48%, respectively. Given that environmental temperature and dehydration may influence the receptor's response, maintaining stability across varying temperature gradients and durations is critical for practical applications. As illustrated in Figure S15c (Supporting Information), the GMTH can stably respond to the pressure stimuli at various temperature gradients, which is attributed to the highly linear voltage and current response of temperature difference. The non‐drying performance endows the GMTH with high durability in long‐term pressure sensing and the sensitivity has a negligible fluctuation when stored in ambient conditions for over 30 days (Figure S15c, Supporting Information). These experiments demonstrate that the GMTH‐based receptor has a high sensitivity and robustness, making it a promising candidate for applications in wearable tactile sensation.

### 2.5 Deep‐Learning‐Assisted Fingertip Receptor for Self‐Supervised Tactile Perception

**Figure 5:**
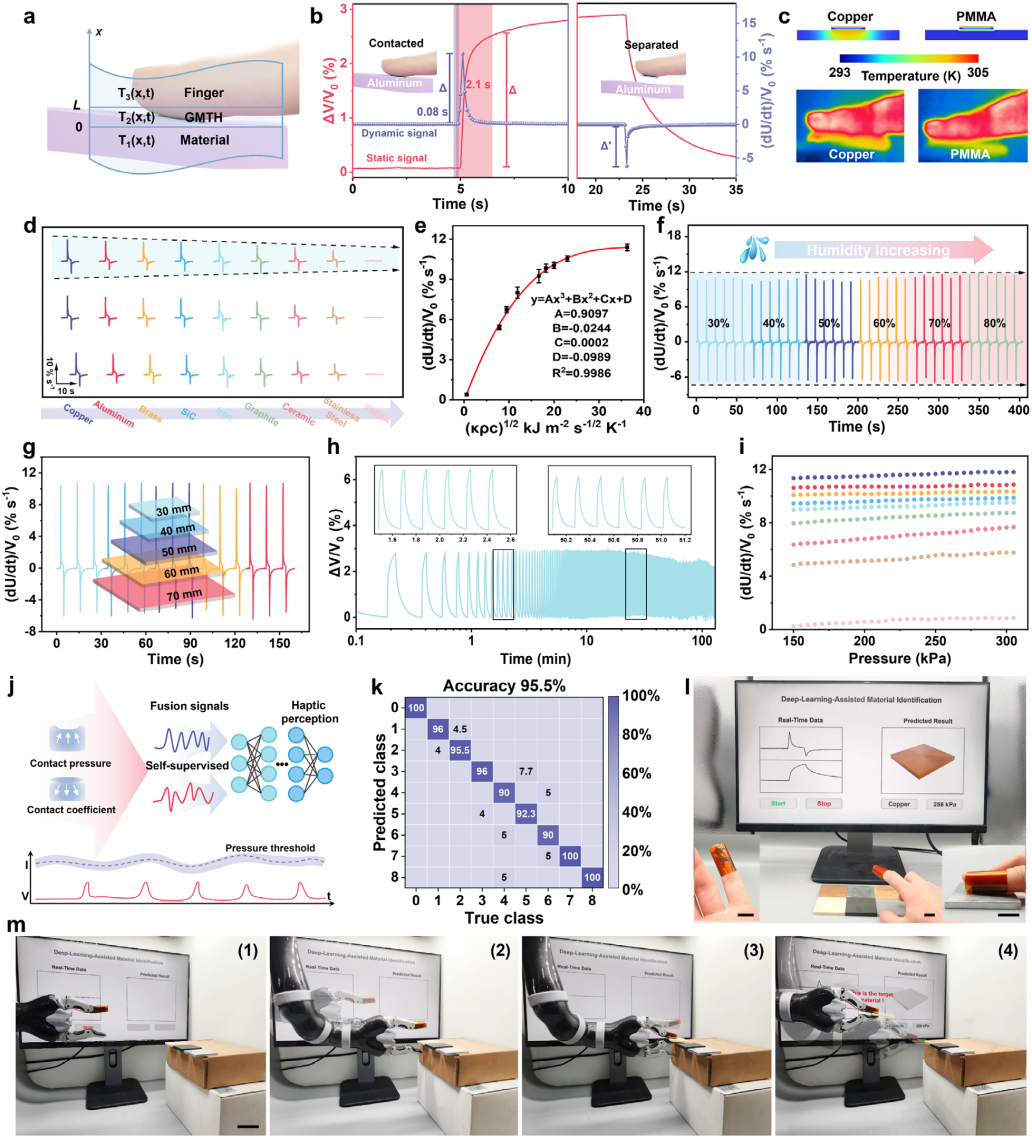
Deep‐learning‐assisted fingertip receptor for self‐supervised tactile perception. a) Schematic diagram of the interfacial heat transfer model. b) Signal characteristics and response time analysis of aluminum detection. c) The temperature distribution in the materials when the receptor contacts the copper and PMMA after 2 s. d) The differential voltage signals of the receptor in response to 9 kinds of materials. e) The universal law of the differential voltage signals as a function of the contact coefficient. f,g) The differential voltage signals of the receptor under different environment humidity and material sizes. h) Fatigue testing of thermoelectricity based on the interfacial heat transfer model of aluminum detection by the fingertip receptor. i) Differential voltage signals of the fingertip receptor under different pressures. The different colored points represent the datasets of different materials and the color order is consistent with Figure 5d. j) The proposed deep learning algorithm for self‐supervised tactile perception by combining pressure and material cues. k) The classification accuracy when the hand wearing the fingertip receptor to touch different objects. Material types correspond to the numbers ranging from 0 to 8. l) Demonstration of the fingertip receptor successfully recognizing the material type with the aid of a deep learning model. Insets are the photo of the receptor and touching process. Scale bar 15 mm. m) Process of the robotic arm performing object recognition with the fingertip receptor and Peltier unit as a heat source. Scale bar 30 mm. 1) The initial stage of object recognition. 2) The robotic arm gradually approaches the test object. 3) The robotic gripper touches the object and identifies it through unsteady interfacial heat conduction. 4) The robotic arm grasps and moves the target object.

Haptic perception is regarded as one of the key functions of human skin. For multimodal receptors, the incorporation of material identification expands the range of tactile perception in biomedical electronics. However, accurately identifying materials with similar smooth surfaces or electronegativity poses a significant challenge. Inspired by the thermoreceptors on neurons, the developed receptor, with the assistance of deep learning, can surpass human skins in material recognition by utilizing thermoelectricity to visualize contact heat transfer variation. The detailed operation principle is schematically shown in **Figure** **5**a and the corresponding theoretical model is established in Note S2 (Supporting Information). The GMTH‐based receptor worn on the finger generates a thermal gradient along the x direction and the thermovoltage is produced between the hot and cold ends owing to thermoelectric conversion. When an object comes into contact with the side of the receptor away from the fingertip, the transient heat conduction from the hydrogel to the object appears, leading to a lower contact surface temperature of the receptor and a larger temperature gradient along the thickness direction of the hydrogel. This process highly resembles the thermal sensation of human skin when touching different materials. With the heat‐to‐electricity conversion of the GMTH, the variation of temperature difference can result in an obvious thermovoltage variation. The signal can be transformed into two parts of static voltage surge and dynamic differentiation voltage spike, which effectively reflect intrinsic cutaneous cues of materials (Figure 5b). The response times of the signals are 2.1 and 0.08 s, respectively, demonstrating the huge potential of the receptor in real‐time object recognition (Figures S16 and S17, Supporting Information).

Thermal contact coefficient is an important thermophysical parameter of materials that affects interfacial heat transfer and energy accumulation in the contact area between the receptor and object. Thanks to the high contact coefficient of the metal, heat can rapidly diffuse to the interior and will not accumulate near the contact surface, leading to an increase in the temperature difference between the two sides of the receptor. In contrast, a greater heat transfer barrier exists along the x direction for the object with a lower contact coefficient, thus the temperature difference is almost constant (Figure 5c). The minimum perceptible temperature gradient of the GMTH is measured as low as 0.1 K (Figure S18a, Supporting Information). As a proof of concept, distinguishable electrical signals are collected under a given pressure of 200 kPa when the receptor contacts nine kinds of materials involving copper, aluminum, iron, PMMA, and so on (Figure 5d; Figure S18b, Supporting Information). The signals clearly demonstrate the discrepancy in both waveforms and amplitudes for each material. This conclusion is systematically validated through theoretical calculations, as presented in Note S2 (Supporting Information). Here, in order to simplify the solution, the receptor and object are both modeled as semi‐infinite bodies and only 1D transient heat transfer along the x direction is considered. According to the above conditions, the thermal diffusivity α_*i*_ is calculated as (*i* = 1: material, *i* = 2: hydrogel, *i* = 3: finger)

(1) $$\alpha_{i}=\frac{k_{i}}{\rho_{i}c_{i}}$$

*k*, ρ, and *c* are the thermal conductivity, density, and specific heat capacity, respectively. The transient heat transfer equation is:

(2) $$\left\{\begin{array}{c} \alpha_{1}\;\frac{\partial^{2}T_{1}\left(x,t\right)}{\partial x^{2}}=\frac{\partial T_{1}\left(x,t\right)}{\partial t}\;\\ \alpha_{2}\;\frac{\partial^{2}T_{2}\left(x,t\right)}{\partial x^{2}}=\frac{\partial T_{2}\left(x,t\right)}{\partial t}\;\\ \alpha_{3}\;\frac{\partial^{2}T_{3}\left(x,t\right)}{\partial x^{2}}=\frac{\partial T_{3}\left(x,t\right)}{\partial t}\; \end{array}\right.$$

where *T*_1_(*x*,*t*), *T*_2_(*x*,*t*) and *T*_3_(*x*,*t*) are the temperature of the material, hydrogel, and finger in the x direction at time t, respectively. When touching an object, the surface temperature of the receptor (*T*_2_) immediately adjusts to the contact temperature (*T*_3_) and *T*_2_ can be expressed as:

(3) $$\begin{array}{ccc} T_{2}\left(x,t\right) & = & J\left\{\frac{1}{K}erfc\left(\frac{x}{2\sqrt{\alpha_{2}t}}\right)\right.\\ & & \left.-\;\frac{1}{K}e^{\left(Kx+\alpha_{2}K^{2}t\right)}erfc\left(\frac{x}{2\sqrt{\alpha_{2}t}}+K\sqrt{\alpha_{2}t}\right)\right\}+T_{20} \end{array}$$

where *J* and *K* are coefficients, $J=-\frac{1}{k_{2}}\left\{\frac{T_{20}-T_{10}}{R}+h\left(T_{30}-T_{20}\right)\right\}$, $K=-\frac{1}{k_{2}}\left\{h+\frac{1}{R}\left(\frac{e_{2}+e_{1}}{e_{1}}\right)\right\}$, $e_{2}=\sqrt{k_{2}\rho_{2}c_{2}}$, $e_{1}=\sqrt{k_{1}\rho_{1}c_{1}}$. *T*_20_ and *T*_30_ are the initial temperature of the receptor and object. In this process, a material's contact coefficient ($\sqrt{k\rho c}$) defines its capability of conducting and extracting heat from the receptor, determining the *T*_2_ at the contact interface. The derivative of temperature with time can be deduced as:

(4) $$\frac{dT}{dt}=\frac{\partial T_{2}\left(x,t\right)}{\partial t}{|}_{x=0}=-J\left[K\alpha_{2}e^{K^{2}\alpha_{2}t}erfc\left(K\sqrt{\alpha_{2}t}\right)-\frac{\sqrt{\alpha_{2}t}}{\sqrt{\pi }t}\right]$$

The Seebeck coefficient (*S_e_*), a constant for the thermogalvanic hydrogel, conveys its ability to convert temperature difference into thermovoltage.

(5) $$\frac{dU}{V_{0}}=\frac{-S_{e}\times dT}{V_{0}}$$

Assuming that ΔV is equal to U(t), its variation as a function of time is given by:

(6) $$\frac{dU}{dt}\times \frac{1}{V_{0}}=\frac{-S_{e}}{V_{0}}\times \frac{dT}{dt}=\frac{S_{e}J}{V_{0}}\left[K\alpha_{2}e^{K^{2}\alpha_{2}t}erfc\left(K\sqrt{\alpha_{2}t}\right)-\frac{\sqrt{\alpha_{2}t}}{\sqrt{\pi }t}\right]$$

where *V*_0_ is a constant thermovoltage related to initial temperature difference between the finger and surroundings. Equation (6) indicates that the rate of voltage change of the hydrogel responds to the contact coefficient *e*_1_ of the target material. It can be concluded that a higher thermal contact coefficient corresponds to a higher $\frac{dU}{dt}$. Based on the Equation (6), we fitted a general function between the maximum rate of voltage change and the thermal contact coefficient of the materials, as depicted in Figure 5e. Consequently, material recognition can be delivered based on the function. In view of the practical tactile perception in an open environment, we have examined the effect of environmental factors on the tactile perception of the receptor. Thanks to the robust interfacial heat transfer process and thermoelectric conversion, the humidity (Figure 5f), object size (Figure 5g), and contact position (Figure S19, Supporting Information) have no obvious interference with thermovoltage signals of the receptor, enabling entropy‐stabilized material fingerprint perception in an open environment. In addition, the thermovoltage response can be stably maintained for over 500 successive cycles of alternately contacting and separating (Figure 5h).

The interfacial heat transfer behaviors observed from the materials are influenced not only by the inherent contact coefficient but also by applied pressure. Once the receptor contacts the same material with different pressures (ranging from 5 to 50 kPa), the amplitude of voltage varies linearly with the applied pressure, which is derived from the more effective heat transfer between the receptor and the detected material (Figure S20, Supporting Information). Therefore, it turns out that monitoring the voltage signal solely is insufficient to accurately recognize the object. To self‐decouple the contact pressure and thermovoltage to enhance the target recognition accuracy, we set a pressure sensing module to monitor the contact pressure upon the GMTH's active piezoresistivity. A PDMS layer is fixed on the top electrode of the pressure module to avoid mutual interference from thermal fluctuation and compressing during contact. As depicted in Figure 5i, the thermovoltage of material recognition changes slightly after applying the pressure above the threshold of 150 kPa. The different colored points represent the datasets of different materials and all the datasets show clear boundaries under various pressures, reducing the difficulty to backend processing and analyzing.

Inspired by the thermoreceptors and mechanoreceptors in human skins, we propose a deep‐learning‐assisted self‐supervised GMTH‐based fingertip receptor integrated with two thermogalvanic hydrogels as an augmented somatosensory receptor to recognize materials and contact pressure by fast and slow adaptive sensing (Figure 5j). The self‐supervised capability can be achieved by analyzing voltage responses of the temperature sensing module at a known pressure measured through the pressure sensing module. Therefore, the object material can be recognized by decoupling the compound dynamic‐static voltage signals based on the referring plot and multimodal feature matrix (Figure 5i; Figure S21, Supporting Information). Initially, the fingertip receptor without the pressure module was mounted on a linear motor with preset pressure and motion protocols to touch different objects. The voltage signals corresponding to nine different materials were collected and subsequently fed into a deep‐learning model for object recognition. Using static voltage signal alone in object classification is insufficient to accurately discriminate objects with similar thermal contact coefficients, resulting in a low classification accuracy of 87.6% (Figure S22, Supporting Information). By contrast, by combining static thermal voltage with dynamic differential voltage, the most characteristic information is extracted, which can improve the accuracy of 96.6% (Figure S23, Supporting Information). As a proof of concept, a fingertip receptor with the temperature and pressure module was worn on the finger to sense materials, and a high recognition accuracy of 95.5% was achieved in a self‐supervised pressure measurement method (Figure 5k; Figure S24, Supporting Information). Furthermore, an immersive tactile perception platform based on the fingertip receptor is designed. The finger wearing the receptor touches the object and the coupled signals related to pressure and materials are transmitted for data analysis and object recognition, which is subsequently displayed on a screen (Figure 5l; Video S1, Supporting Information). To demonstrate the versatility of the proposed fingertip receptor, we integrated the receptor on a robotic arm to achieve target material recognition and grasping. To imitate the constant temperature of fingers, a Peltier unit is located between the receptor and the robotic gripper. With the help of the self‐supervised receptor, the robotic arm can successfully identify different objects and perform reliable grasping (Figure 5m; Video S2, Supporting Information). As a result, this receptor breaks through the bottleneck of common tactile perception, whose signals are usually affected severely by contact pressure and open environments.

## 3 Conclusion

This study presents a novel fast and slow adaptive sensing strategy to mimic human tactile perception and quantify somatosensory parameters using a synergistic combination of thermogalvanic and active piezoresistive effects. By combining the elaborately created micropatterned and gradient structure, the thermogalvanic hydrogel demonstrates a maximum pressure sensitivity of 53.6 kPa^−1^ and a low detection limit of 1.9 Pa. Benefiting from the combing of static and dynamic signals, rapid identification of different materials within 80 ms is delivered by utilizing the thermoelectricity to visualize contact heat transfer variation. The accuracy of recognizing materials is independent of the applied contacting force and the thermovoltage maintains stability under various external surroundings and contact conditions. On the basis of the above mechanism, we developed a wearable and self‐powered multimodal fingertip receptor with augmented human tactile perception to perceive the object material in a self‐supervised mode. With the assistance of a pre‐trained deep‐learning algorithm, the material recognition accuracy can reach as high as 95.5%. Furthermore, a real‐time object recognition based on the fingertip receptor was demonstrated, which offers a compelling solution for assisting patients with sensation disorders and robotic arms to perception. In the future, exploring additional test samples and miniaturized sensing platforms is essential for broader practical applications.

## 4 Experimental Section

### Materials

PVA (molecular weight, 120,000 to 140,000), potassium acetate (KAc, ≥99.0%), Dimethyl Sulfoxide (DMSO, ≥99.5%), K_4_Fe(CN)_6_ (MW = 329.2, ≥99%), K_3_Fe(CN)_6_·3H_2_O (MW = 422.4, ≥99%) were purchased from Sigma–Aldrich. Polyimide (PI, 50 µm) film was purchased from an online shop. All materials were used as received without further purification.

### Preparation of the Receptor

The thermogalvanic hydrogels with micro‐pyramid and gradient modulus were fabricated via two steps. First, 10 wt.% PVA was dissolved in a mixture of 14 mL DMSO and 6 mL deionized water with stirring 2 h at 800 rpm until a transparent and homogeneous solution was yielded. Afterward, the obtained solution was poured into a PDMS mold and stored at 253 K for 24 h. Finally, the produced hydrogel was placed into an open mold with the bottom wrapped by a dialysis membrane, which was dialyzed in 100 mL of different KAc solutions at various temperatures for a certain time (Figure S1a, Supporting Information). The LIG electrodes were prepared by a CO_2_ laser with a wavelength of 10.6 µm and a spot size of 130 µm in the engraving mode. To improve the hydrophilicity, the electrodes were soaked in 30 wt.% hydrochloric acid solution for 12 h. Subsequently, the electrodes were thoroughly washed with the deionized water and dried in the air.

### Assembly of Fingertip Receptor

The fingertip receptor was fabricated in a fingertip‐like configuration to ensure conformal integration with the human finger, enabling reliable and stable tactile perception during natural movements. The sensing unit comprises two circular thermogalvanic hydrogel elements that serve as the mechanoreceptor and thermoreceptor, respectively. Each hydrogel block is 1 mm thick with a diameter of 10 mm. Laser‐induced graphene (LIG) films patterned on polyimide (PI) substrates were employed as both the electrodes and the structural encapsulation layers. The LIG electrodes offer excellent flexibility and conductivity, while the PI substrate ensures mechanical durability. During assembly, two LIG/PI films were laminated with the thermogalvanic hydrogel elements sandwiched between them, and the entire multilayer structure was subsequently rolled into a cylindrical geometry that conforms to the curvature of a human fingertip. To secure the rolled structure, adhesive tape was applied along the interface where the two PI layers meet, forming a stable, wearable fingertip‐shaped device. This integrated structure supports multimodal sensing while maintaining mechanical flexibility and conformability. The overall device architecture is illustrated in Figure 1e.

### Material Characterization

The morphology and chemical compositions of the hydrogels were characterized by SEM (Hitachi SU8010) and FTIR (Spectrum 100). The thermal stability was evaluated using the thermogravimetric analyzer (TGA‐601, HUIC) under an air atmosphere with a scan rate of 20 °C min^−1^. The tensile and compressive mechanical tests were conducted by a universal mechanical test machine (QT‐1196) under ambient conditions (293 K, 30% RH). The transmittance of the hydrogel was tested by employing a UV–vis spectrometer (Shimadzu UV‐1780). The hydrophilicity of the LIG electrode was performed on the contact angle measuring instrument (OCA20, America) at room temperature. Wide‐angle X‐ray scattering (WAXS) measurements (Xeuss 2.0) with a wavelength of 0.154 nm were conducted to estimate the number and average size (D) of crystalline domains based on the Scherrer equation: D = kλ/(βcosθ).

### Electrical Performance Characterization

The thermoelectric output was collected by a Keithley 2400 source meter and the temperature difference was controlled by the Peltier chips and recorded by thermocouples (NAPUI TR230X) under ambient conditions (293 K, 30% RH). The electrochemical impedance spectroscopy (EIS) and cyclic voltammetry (CV) of the hydrogels were measured by an electrochemical workstation (CHI660e, Shanghai Chenhua Instrument Co. Ltd.). Non‐drying performance tests were performed at 293 K and 60% RH. For material recognition experiments, the relative humidity was varied from 30% to 80% at 293 K to evaluate sensing stability under different humidity levels. Other contact conditions experiments, including contact position and material size, were also carried out at 293 K and 30% RH. A commercial linear motor (LinMot‐E1200) was utilized in the material data acquisition process.

### Training and Optimization of the Deep Learning Model

The multimodal sensing signals of the fingertip receptor were sampled by a multichannel data collector and delivered to the computer for subsequent signal feature extraction via the USB interface. The structure of the deep learning model is depicted in Figure S21 (Supporting Information). The pressure and material signals were processed by the 1D convolution and Resnet‐34 to extract features, respectively. Then, the obtained feature maps were concatenated and input to a multilayer perceptron (MLP) for self‐supervised materials classification. During the training process, the data sets of recognition voltage including 9 kinds of objects were collected. 75% of the voltage samples were randomly chosen from the database for training, with the remaining 25% designated as test samples. The customized interactive interface was programmed by Pytorch library and operated on a laptop. When performing tactile perception tasks, the interface calls the trained MLP model developed by Python and sends the real‐time voltage/current signals to the MLP model for identifying the object.

## Conflict of Interest

The authors declare no conflict of interest.

## Author Contributions

H.Z. and Z.‐H.L. conceived the research idea and supervised the entire project. N.L., Z.W., and Y.N. designed and carried out the experiments. N.L., Y.L., and S.W. analyzed the results and wrote the manuscript. All the authors discussed the results and commented on the manuscript.

## Supporting information



Supporting Information

Supplemental Video 1

Supplemental Video 2

## Data Availability

The data that support the findings of this study are available from the corresponding author upon reasonable request.
