# Supplementary material for: Bionic Multimodal Augmented Somatosensory Receptor Enabled by Thermogalvanic Hydrogel
Source: Adv Sci (Weinh). 2025 Jun 29;12(32):e05873. doi: 10.1002/advs.202505873 (PMC12407276; doi:10.1002/advs.202505873)
Supplement: Supplementary file 1 — Supporting Information [file ADVS-12-e05873-s001.docx]

**Supplementary Materials**

**Bionic multimodal augmented somatosensory receptor enabled by thermogalvanic hydrogel**

*Ning Li, Zhaosu Wang,* *Yu Niu, Yu Li, Suyi Wen, Hulin Zhang*,* *Zong-Hong Lin**

Ning Li, Zhaosu Wang, Yu Niu, Hulin Zhang

College of Integrated Circuits, Taiyuan University of Technology, Taiyuan, 030024, China

E-mail: zhanghulin@tyut.edu.cn

Yu Li, Suyi Wen

College of Electronic Information Engineering, Taiyuan University of Technology, Taiyuan, 030024, China

Zong-Hong Lin

Department of Biomedical Engineering, National Taiwan University, Taipei, 10167, Taiwan

E-mail: zhlin@ntu.edu.tw

**Supplementary Text**

**Note S1. Thermal conductivity measurement**

As shown in Figure 3d, the thermal conductivity (κ) of the GMTH was measured by a steady-state method [1,2]. The measurement device consists of two identical poly(methyl methacrylate) (PMMA) plates (a thickness ($d$) of 4 mm and a thermal conductivity (κ_1_, κ_3_) of 0.19 W m^-1^ K^-1^) in parallel at the both sides of the GMTH as heat transfer walls, with a distance of 4 mm. A Peltier heating unit contacts the left PMMA wall. The GMTH with a thermal conductivity of κ_2_ is placed between two PMMA plates and the cross-sectional area of the GMTH is 4 cm^2^ (20×20 mm). In order to ensure that the input thermal flow (𝑄_𝑖𝑛𝑝𝑢𝑡_) is equal to the output thermal flow, the whole device is embedded in insulation foam to prevent heat dissipation into the surroundings. Therefore, the heat flow across the two plate walls is equal to the heat flow across the middle hydrogel, which is defined as:

$$\begin{aligned} \kappa_{1}A\left( \frac{\partial T}{\partial d} \right)_{1}=\kappa_{2}A\left( \frac{\partial T}{\partial d} \right)_{2}=\kappa_{1}A\left( \frac{\partial T}{\partial d} \right)_{3}\#\left( 1-1 \right) \end{aligned}$$

$$\begin{aligned} \kappa_{2}=\kappa_{1}\frac{\left( \frac{\partial T}{\partial d} \right)_{1}}{\left( \frac{\partial T}{\partial d} \right)_{2}}=\kappa_{1}\frac{\left( \frac{\partial T}{\partial d} \right)_{3}}{\left( \frac{\partial T}{\partial d} \right)_{2}}\#\left( 1-2 \right) \end{aligned}$$

According to Equation (1-1), if the steady-state temperature gradient (∂T/∂d) is achieved across the PMMA walls and hydrogel, then we can calculate the thermal conductivity of the hydrogel. The temperature changes for the hydrogel and PMMA walls was monitored using thermocouples (NAPUI TR230X). For each measurement, the device was incubated for a sufficient time (>1 h) to ensure that a steady temperature gradient has been built.

**Note S2. Theoretical analysis of thermal contact coefficient sensing mechanism**

In this work, the human body can be considered as a heat source unit. However, to get ideal and stable recognition results in the experiment, the initial temperature gradient of receptor is generated by the Peltier unit (PU). When an object comes into contact with the side of receptor away from the fingertip, the transient heat conduction from the hydrogel to the object appears, leading to a lower contact surface temperature of the receptor and a larger temperature gradient along the thickness direction of the hydrogel. The temperature gradient varies with different thermal contact coefficients of materials. During the stage of heat transfer, the PU, hydrogel and test object can be regarded as semi-infinite models and only one-dimensional heat transfer along the direction perpendicular to the contact surface is taken into account [3-5].

In order to simplify the theoretical model, we consider the thermal resistance between the hydrogel and material, while ignoring the thermal resistance between the hydrogel and PU. According to the above conditions, the model shown in Figure 5a is established. Thermal diffusivity is calculated as ($i$=1: material, $i$=2: hydrogel, $i$=3: PU):

$$\begin{aligned} \alpha_{i}=\frac{k_{i}}{\rho_{i}c_{i}}\#\left( 2-1 \right) \end{aligned}$$

$k$, $\rho$, $c$ are thermal conductivity, density, and specific heat capacity, respectively, which is inherent thermal properties of the objects. The transient heat transfer equation is:

$$\begin{aligned} \left\{ \begin{aligned} \alpha_{1}\frac{\partial^{2}T_{1}\left( x,t \right)}{\partial x^{2}}=\frac{\partial T_{1}\left( x,t \right)}{\partial t} \\ \alpha_{2}\frac{\partial^{2}T_{2}\left( x,t \right)}{\partial x^{2}}=\frac{\partial T_{2}\left( x,t \right)}{\partial t}\#\#\#\#\# \\ \alpha_{3}\frac{\partial^{2}T_{3}\left( x,t \right)}{\partial x^{2}}=\frac{\partial T_{3}\left( x,t \right)}{\partial t} \end{aligned} \right.\#\left( 2-2 \right) \end{aligned}$$

where $T_{1}(x,t)$, $T_{2}(x,t)$ and $T_{3}(x,t)$ are the temperature of the material, hydrogel and PU in the x-plane at time t, respectively. $\alpha_{1}$, $\alpha_{2}$ and $\alpha_{3}$ are the thermal diffusivity of the material, hydrogel, and PU, respectively.

The initial conditions are:

$$\begin{aligned} \left\{ \begin{aligned} T_{1}\left( x,0 \right)=T_{10} \\ T_{2}\left( x,0 \right)=T_{20} \\ T_{3}\left( x,0 \right)=T_{30} \end{aligned} \right.\#\left( 2-3 \right) \end{aligned}$$

Because the contact surfaces of the PU, hydrogel and test objects maintains a continuous heat flow and temperature continuity, it follows as below:

$$\begin{aligned} \left\{ \begin{aligned} k_{3}A\frac{\partial T_{3}}{\partial x}|_{x=L}=k_{2}A\frac{\partial T_{2}}{\partial x}|_{x=L} \\ T_{3}\left( L,t \right)=T_{2}\left( L,t \right) \\ -k_{2}A\frac{\partial T_{2}}{\partial x}|_{x=0}+h\left( T_{30}-T_{2}\left( 0,t \right) \right)=k_{1}A\frac{\partial T_{1}}{\partial x}|_{x=0}=\frac{T_{2}\left( 0,t \right)-T_{1}\left( 0,t \right)}{R} \end{aligned} \right.\#\left( 2-4 \right) \end{aligned}$$

As $x\to\infty$, we typically assume that the temperature of materials and PU remains constant,

$$\begin{aligned} \left\{ \begin{aligned} -k_{2}\frac{\partial T_{3}}{\partial x}|_{x=\infty}=0 \\ -k_{1}\frac{\partial T_{1}}{\partial x}|_{x=\infty}=0 \end{aligned} \right.\#\left( 2-5 \right) \end{aligned}$$

Combining equation (2-2) to (2-5), the temperature profile of the side of the hydrogel away from the PU can be expressed as:

$$\begin{aligned} T_{2}\left( x,t \right)=J\left\{ \frac{1}{K}erfc\left( \frac{x}{2\sqrt{\alpha_{2}t}} \right)-\frac{1}{K}e^{\left( Kx+\alpha_{2}K^{2}t \right)}erfc\left( \frac{x}{2\sqrt{\alpha_{2}t}}+K\sqrt{\alpha_{2}t} \right) \right\}+T_{20}\#\left( 2-6 \right) \end{aligned}$$

$$\begin{aligned} \frac{\partial T_{2}\left( x,t \right)}{\partial t}=-J\left[ K\alpha_{2}e^{K^{2}\alpha_{2}t+Kx}erfc\left( \frac{K\alpha_{2}t}{\sqrt{\alpha_{2}t}}+\frac{x}{2\sqrt{\alpha_{2}t}} \right)-\frac{\sqrt{\alpha_{2}t}e^{-\frac{x^{2}}{4\alpha_{2}t}}}{\sqrt{\pi}t} \right]\#\left( 2-7 \right) \end{aligned}$$

where $J$ and $K$ are coefficients, $J=-\frac{1}{k_{2}}\left\{ \frac{T_{20}-T_{10}}{R}+h(T_{30}-T_{20}) \right\}$, $K=-\frac{1}{k_{2}}\left\{ h+\frac{1}{R}\left( \frac{e_{2}+e_{1}}{e_{1}} \right) \right\}$, $e_{2}=\sqrt{{k_{2}\rho}_{2}c_{2}}$, $e_{1}=\sqrt{{k_{1}\rho}_{1}c_{1}}$. Given our focus on the temperature at the contact point over time, we can disregard the spatial x-dependent component and concentrate solely on solving the time-dependent part. A derivative solution is

$$\begin{aligned} \frac{dT}{dt}=\frac{\partial T_{2}\left( x,t \right)}{\partial t}|_{x=0}=-J\left[ K\alpha_{2}e^{K^{2}\alpha_{2}t}erfc\left( K\sqrt{\alpha_{2}t} \right)-\frac{\sqrt{\alpha_{2}t}}{\sqrt{\pi}t} \right]\#\left( 2-8 \right) \end{aligned}$$

The Seebeck coefficient ($S_{e}$), a constant for the thermogalvanic hydrogel, conveys its ability to convert temperature differences into thermovoltage.

$$\begin{aligned} \frac{dU}{V_{0}}=\frac{-S_{e}\times dT}{V_{0}}\#\left( 2-9 \right) \end{aligned}$$

Assuming that ΔV is equal to U(t), its variation as a function of time is given by

$$\begin{aligned} \frac{dU}{dt}\times\frac{1}{V_{0}}=\frac{{-S}_{e}}{V_{0}}\times\frac{dT}{dt}=\frac{S_{e}J}{V_{0}}\left[ K\alpha_{2}e^{K^{2}\alpha_{2}t}erfc\left( K\sqrt{\alpha_{2}t} \right)-\frac{\sqrt{\alpha_{2}t}}{\sqrt{\pi}t} \right]\#\left( 2-10 \right) \end{aligned}$$

Where $V_{0}$ is a constant thermovoltage related to initial temperature difference between the fingers and surroundings. Equation (2-10) indicates that the rate of voltage change of the hydrogel responds to the contact coefficient $e_{1}$ of the target material.


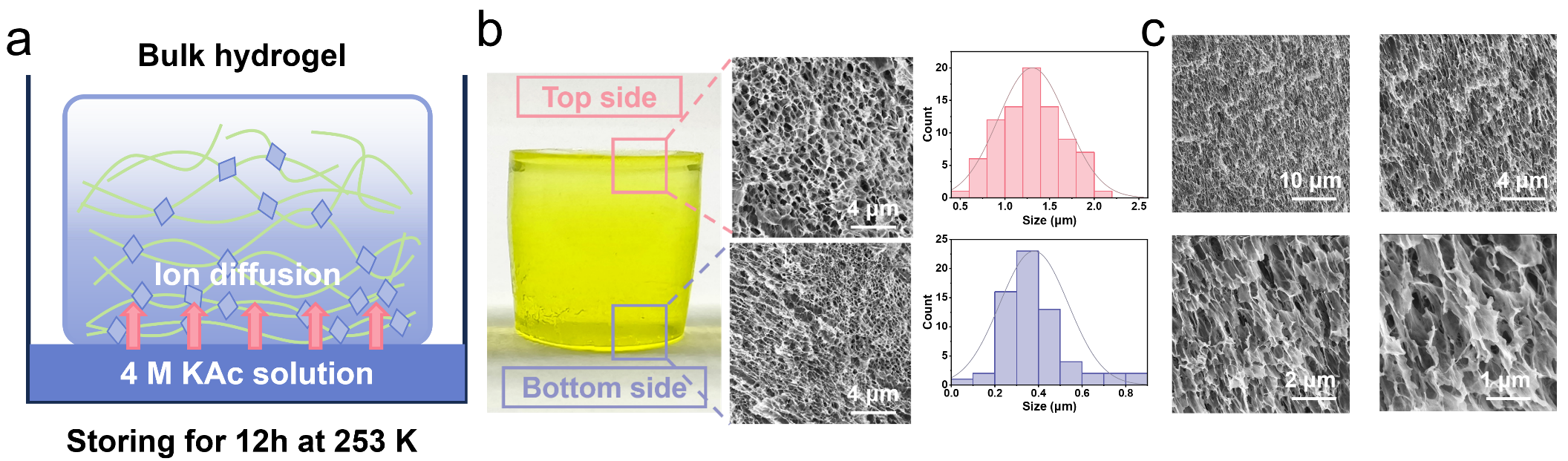


**Figure S1.** (a) Schematic illustration of the preparation process of gradient hydrogels by ion directional diffusion under low temperature. (b) The photo of the gradient hydrogel (left) and the cross-section SEM images as well as the top and bottom pore size distributions (right). (c) The SEM images of the homogenous hydrogel and corresponding magnified images.


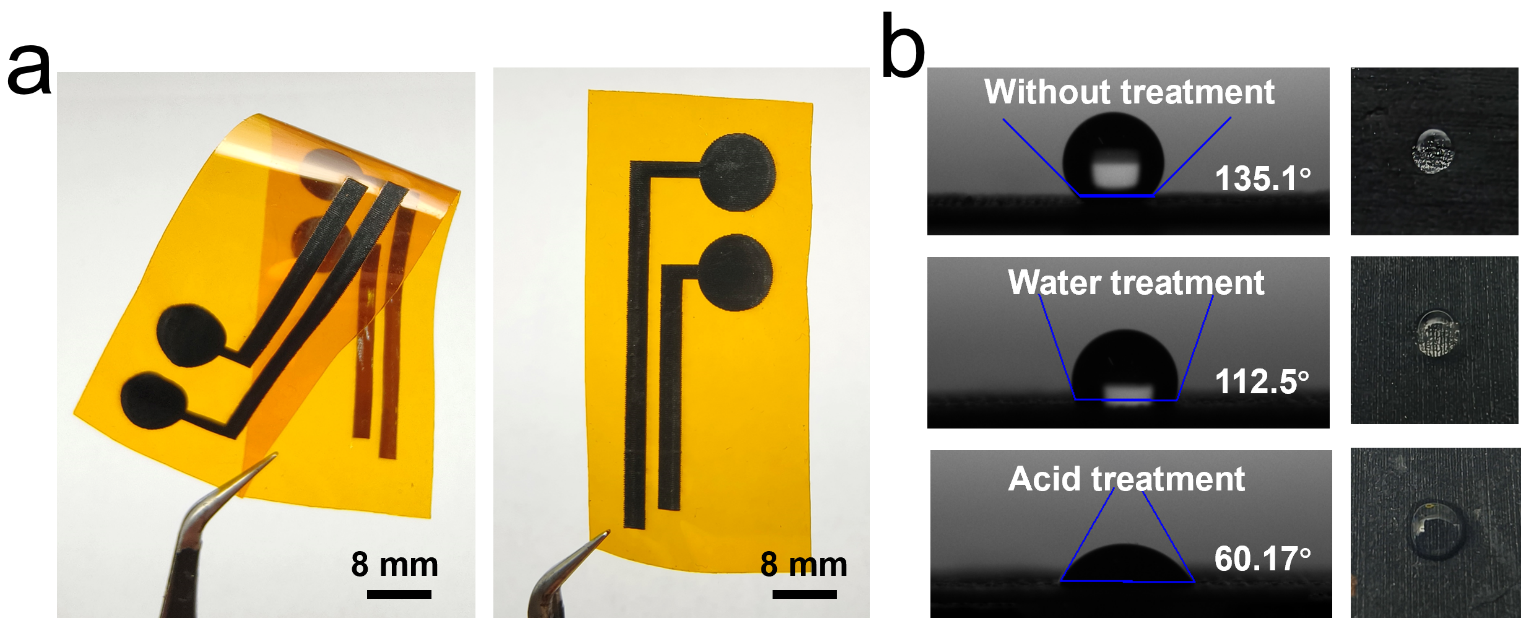


**Figure S2.** (a) The photo of the LIG electrodes. Scale bar 8 mm. (b) The contact angle changes of the LIG electrodes after immersing in deionized water and 30 wt% hydrochloric acid for 12 h.


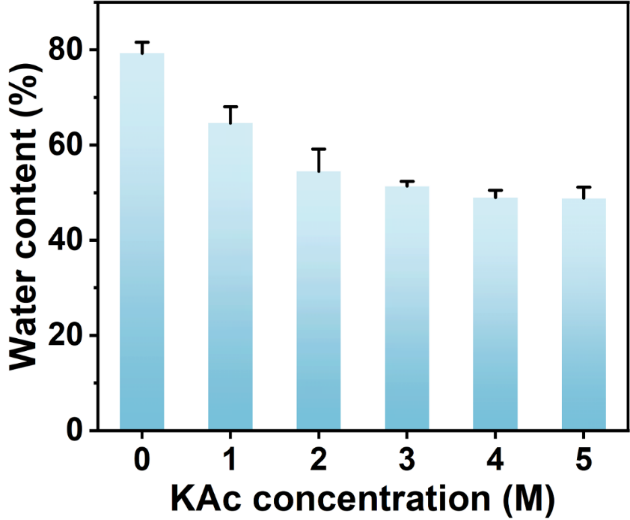


**Figure S3.** Water contents of the hydrogels after immersion in different KAc concentrations.


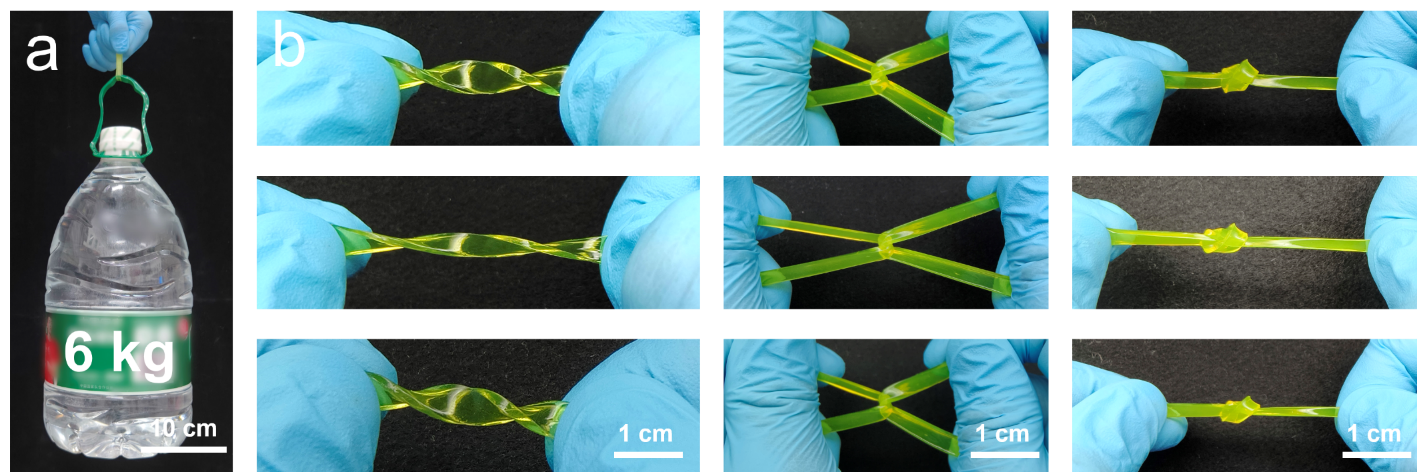


**Figure S4.** (a) Demonstration of the extremely high fracture strength of the GMTH. Scale bar 10 cm. (b) Photographs showing the GMTH exhibits the ability to withstand twisting, cross stretching, and knotting. Scale bar 1 cm.


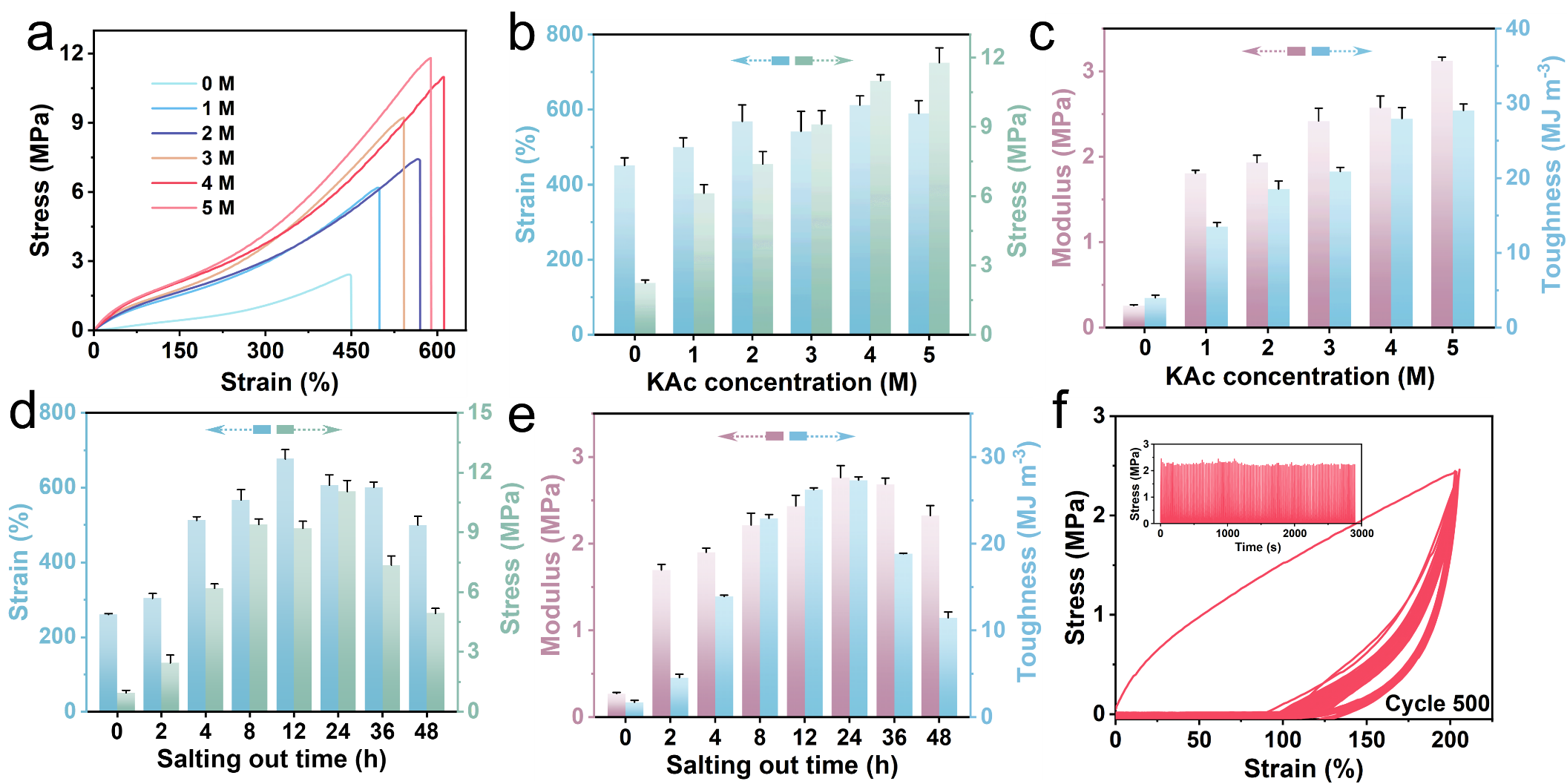


**Figure S5.** (a-c) Tensile performance regulation of the hydrogels by immersion in different concentrations of KAc solutions. (d,e) The effect of soaking duration on the tensile performance. (f) Successive cyclic tensile loading-unloading curves at 200% strain.


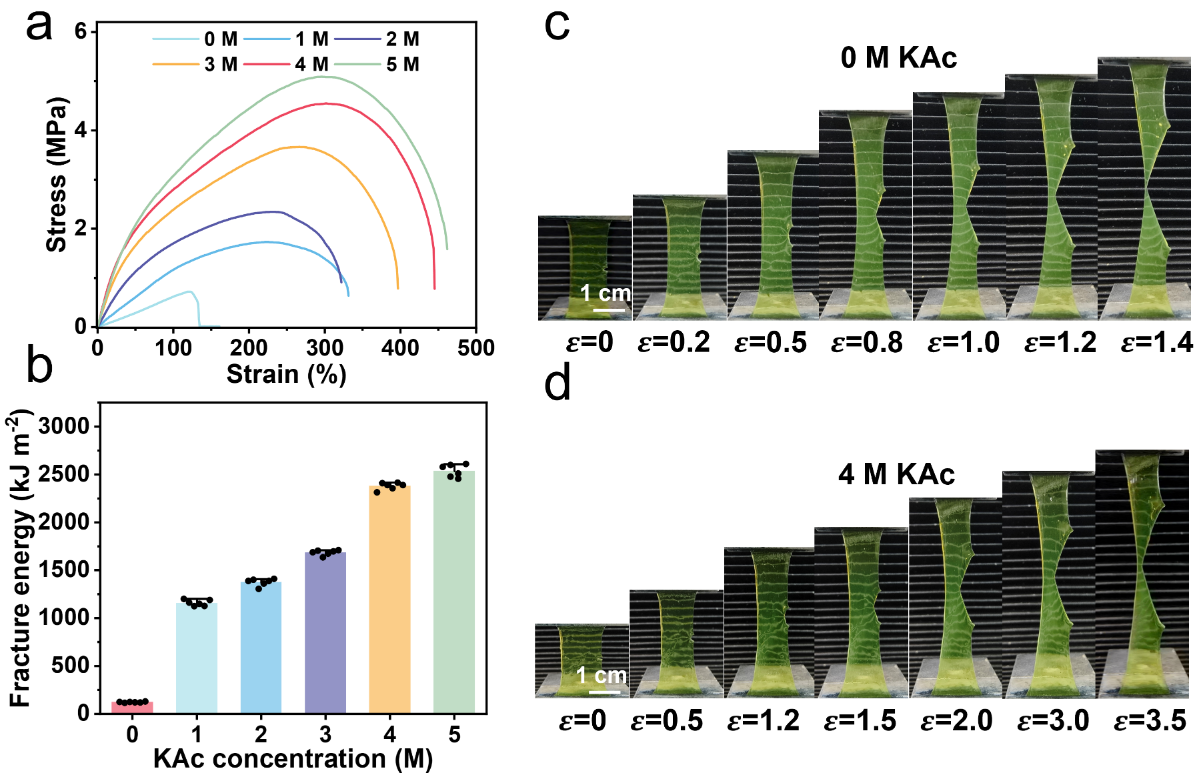


**Figure S6.** (a,b) The stress-strain curves and fracture energy of the notched hydrogels after soaking in various concentrations of KAc solutions. (c,d) Validation of the crack blunting and pinning effects in the GMTH. Scale bar 1 cm.


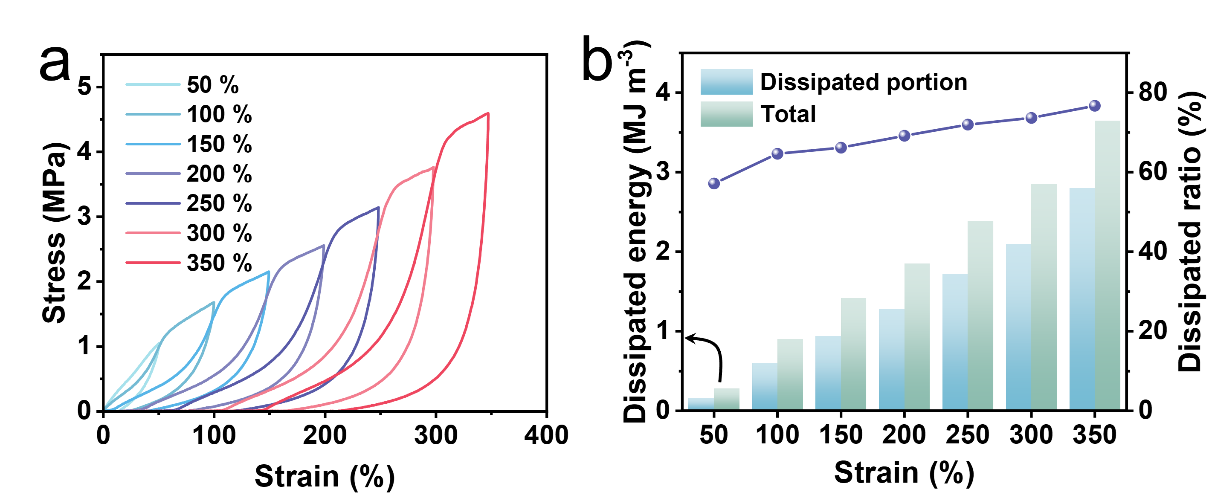


**Figure S7.** (a) The loading-unloading curves at various tensile strain and (b) corresponding total energy and dissipated energy under different strains.


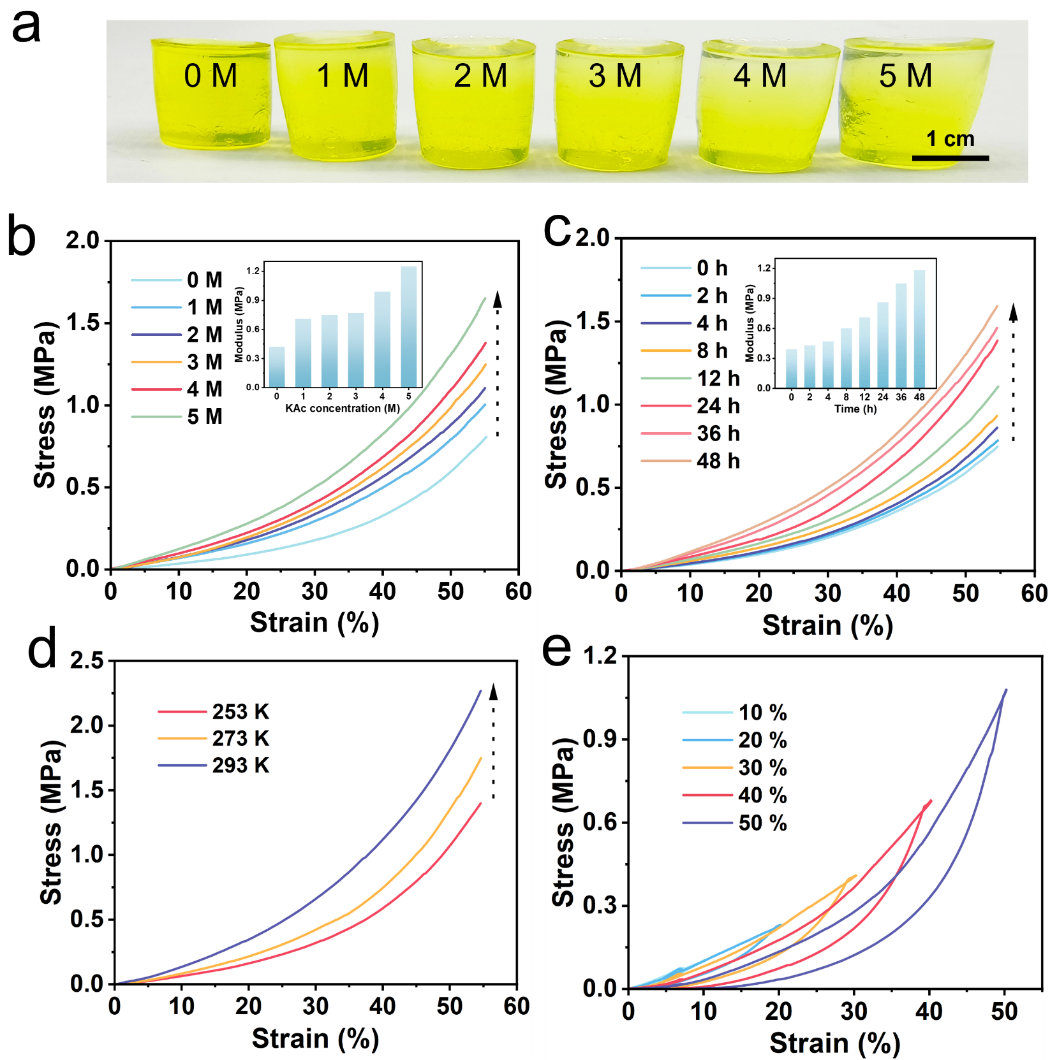


**Figure S8.** (a) The photos showing the hydrogels after soaking in different concentrations of KAc solutions for 12 h. Scale bar 1 cm. (b-d) Compressive performance regulation by changing the immersion concentration, duration, and temperature, respectively. (e) Cyclic compressing curves of the hydrogels under different strains.


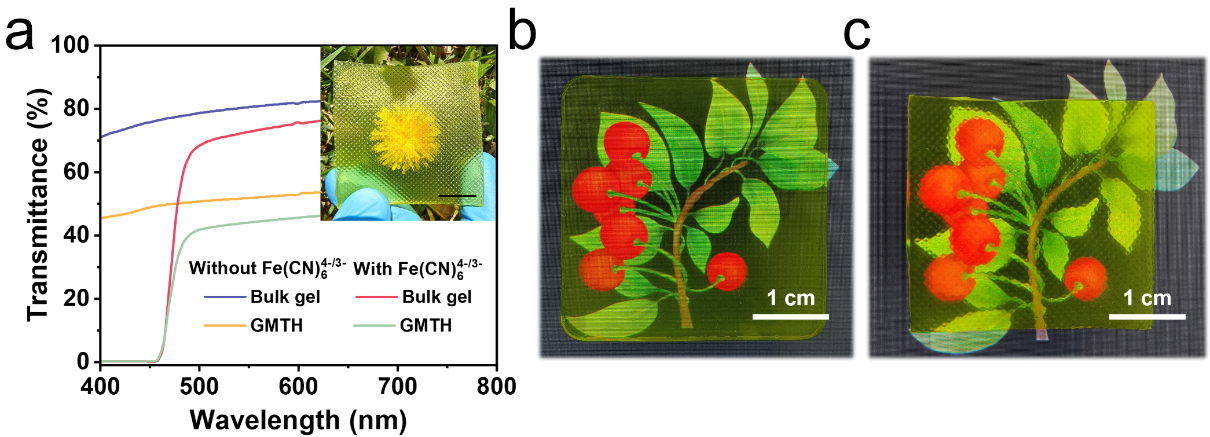


**Figure S9.** (a) Transmittance spectra of the hydrogels with different components, inset photo is the GMTH above a flower. Scale bar 1 cm. (b,c) The optical photos demonstrating the high transparency of hydrogel without and with micro-pyramid. Scale bar 1 cm.


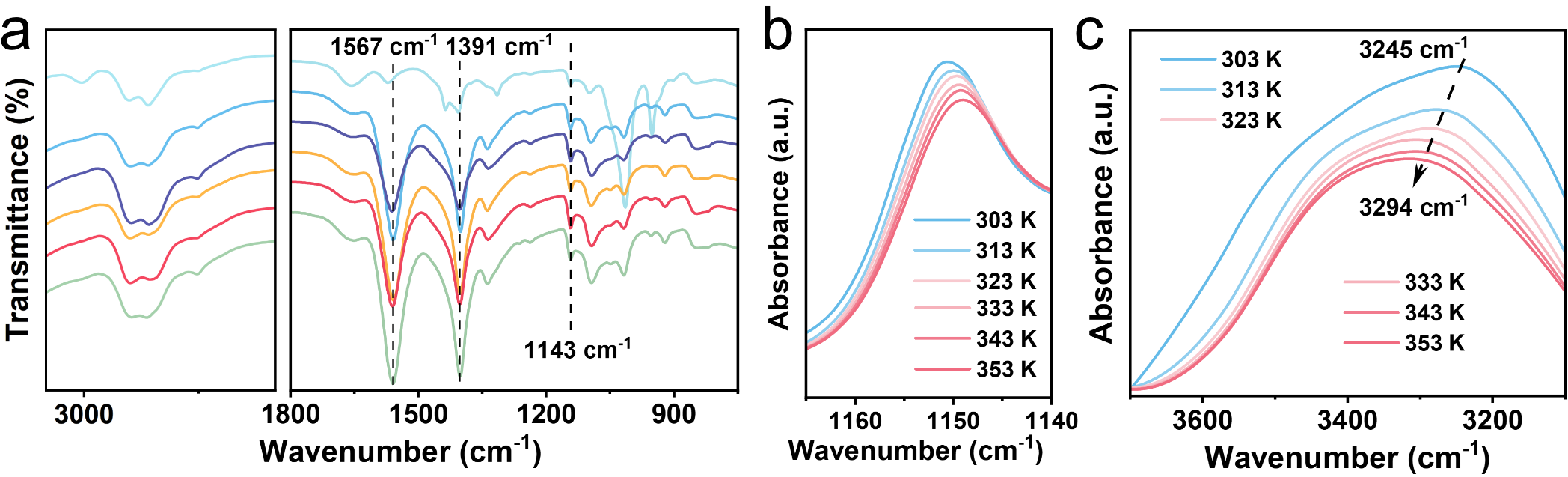


**Figure S10.** (a) FTIR spectra from top to bottom are hydrogels with different KAc concentrations from 0 to 5 M, respectively. (b,c) Temperature-dependent FTIR spectra of the GMTH.


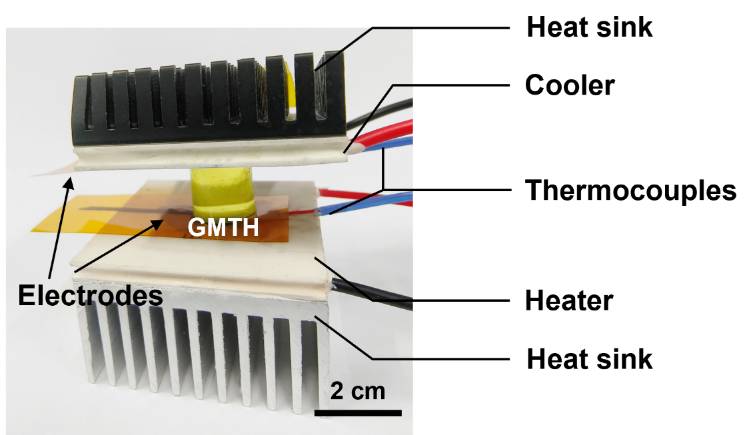


**Figure S11.** Thermoelectric measurement device for electric performance evaluation of the thermogalvanic hydrogels. Scale bar 2 cm.


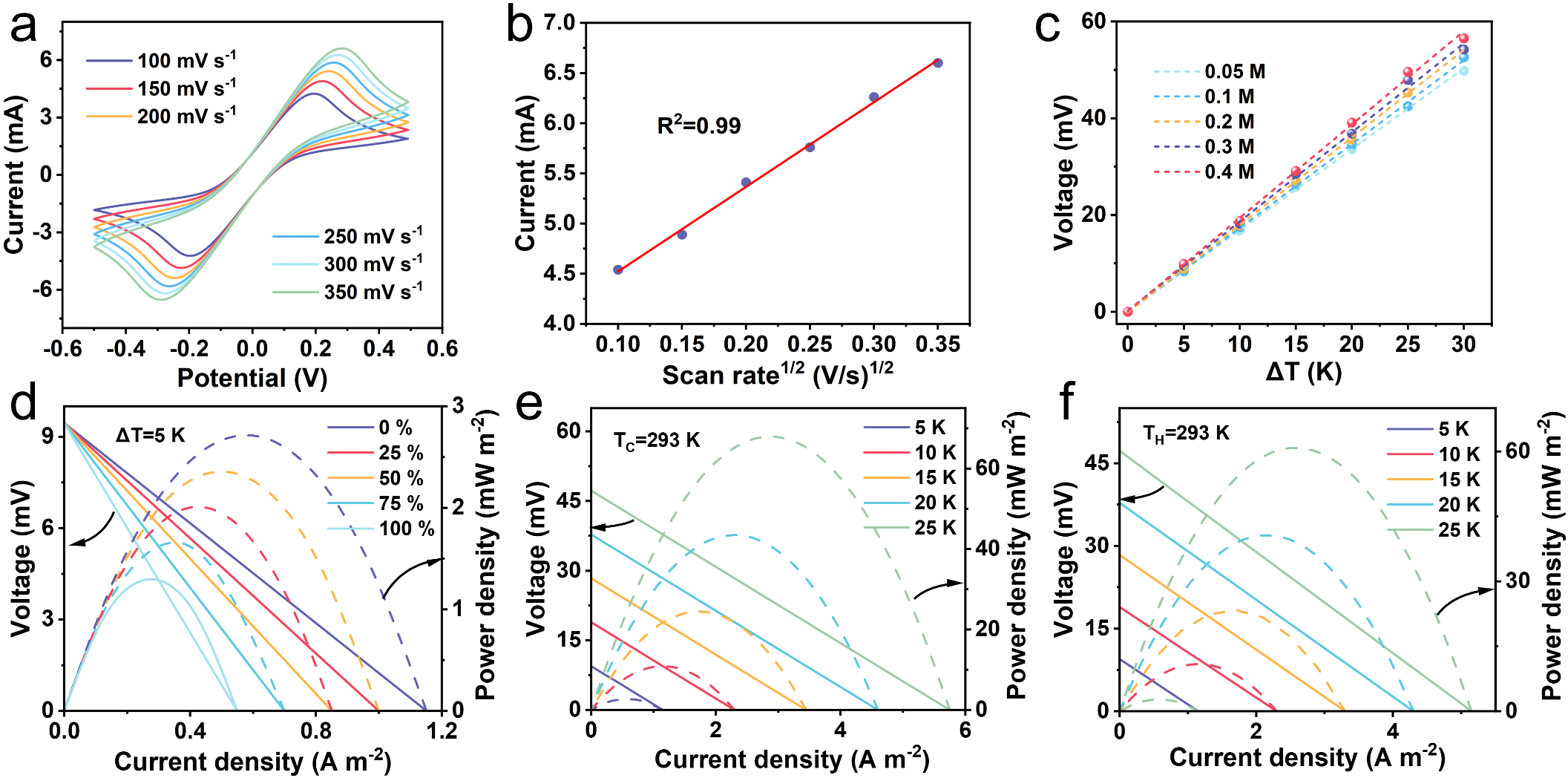


**Figure S12.** (a) CV curves scanned at different rate. (b) Peak current versus square-rooted scan rate. (c) The thermopower measurement results with various [Fe(CN)_6_]^4-/3-^ concentrations. (d) The output voltage-current-power curves at different stretchable strains under the ΔT of 5 K. (e,f) The output voltage-current-power curves under different temperature differences (ΔT) with the temperature of cold side and hot side fixed at 293 K, respectively.


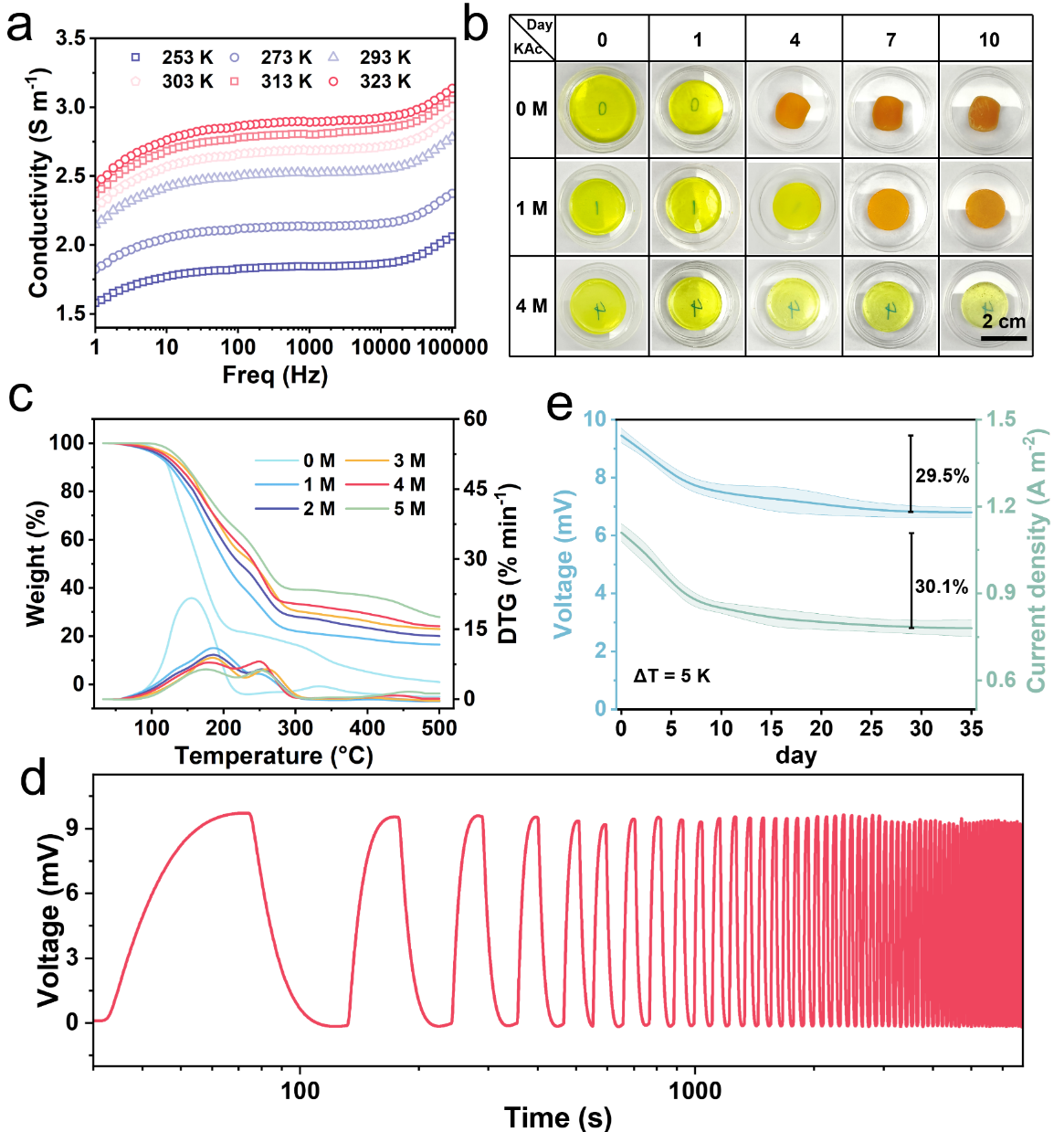


**Figure S13.** (a) Frequency dependent ionic conductivity of the GMTH at different temperatures. (b) Comparison of the hydrogel with different KAc concentrations after being stored for 10 days at 293 K. (c) Thermogravimetric characterization of the hydrogel with different KAc concentrations. (d) Voltage output of the hydrogel over 2h of under applied temperature difference. (e) Electrical performance variation of the GMTH as function of time at 25 ℃ and 40% humidity.


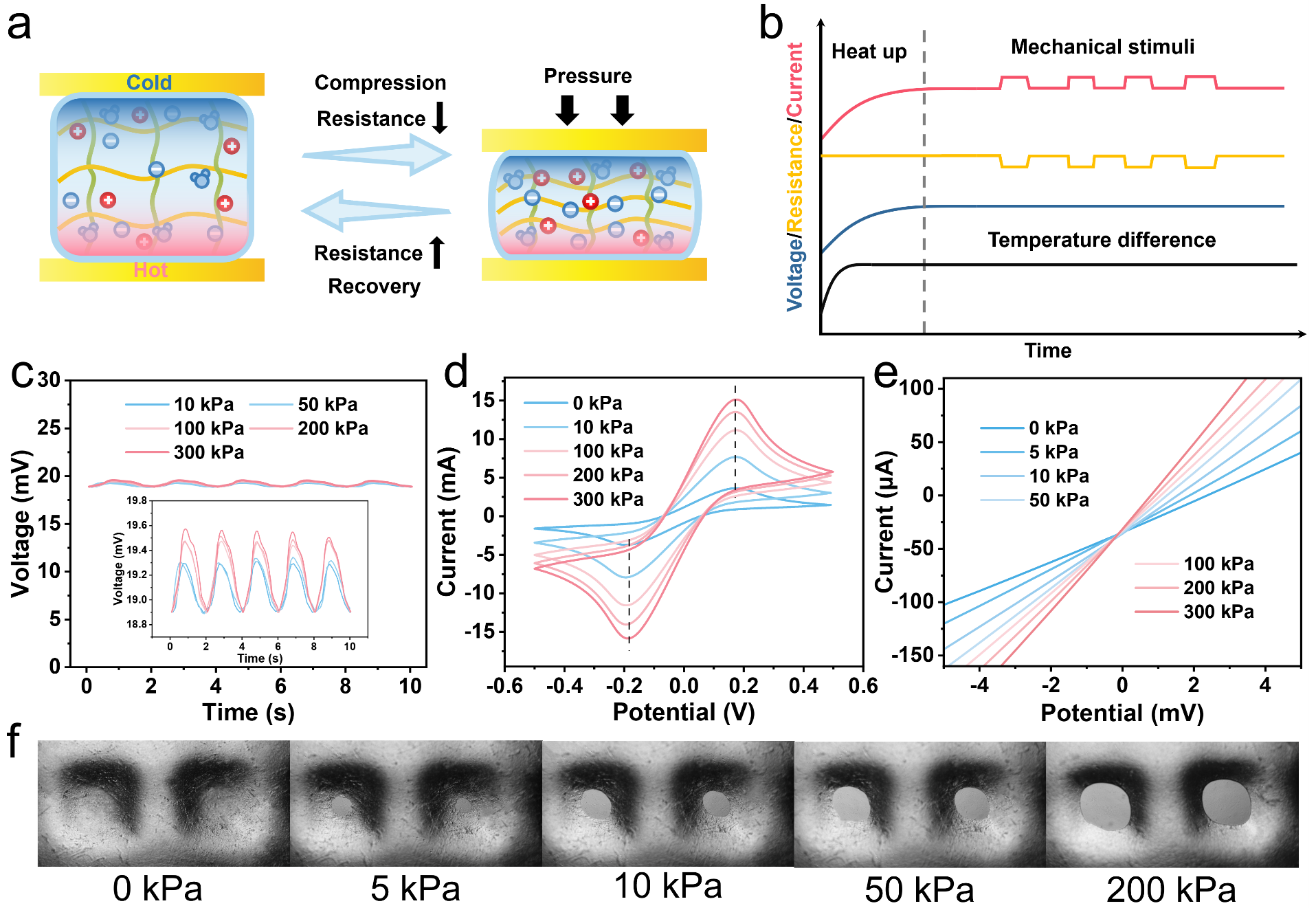


**Figure S14.** (a,b) The active piezoresistivity of the thermogalvanic hydrogel during the compressing and recovering process. In a steady state, a temperature difference is established between the two ends of the hydrogel and an initial voltage is produced based on the thermoelectricity. As the pressure increases, the voltage exhibits pressure insensitivity but the resistance decreases correspondingly, leading to an increased electrical current signal in a self-powered manner. (c) The output voltage of the TGH under different compressing states. Inset shows the enlarged voltage variation. (d) The stability of the redox reactions under different pressures. (e) The I-V curves of the GMTH under different stress. (f) Contact conditions between the microstructure and the electrode under different pressures.


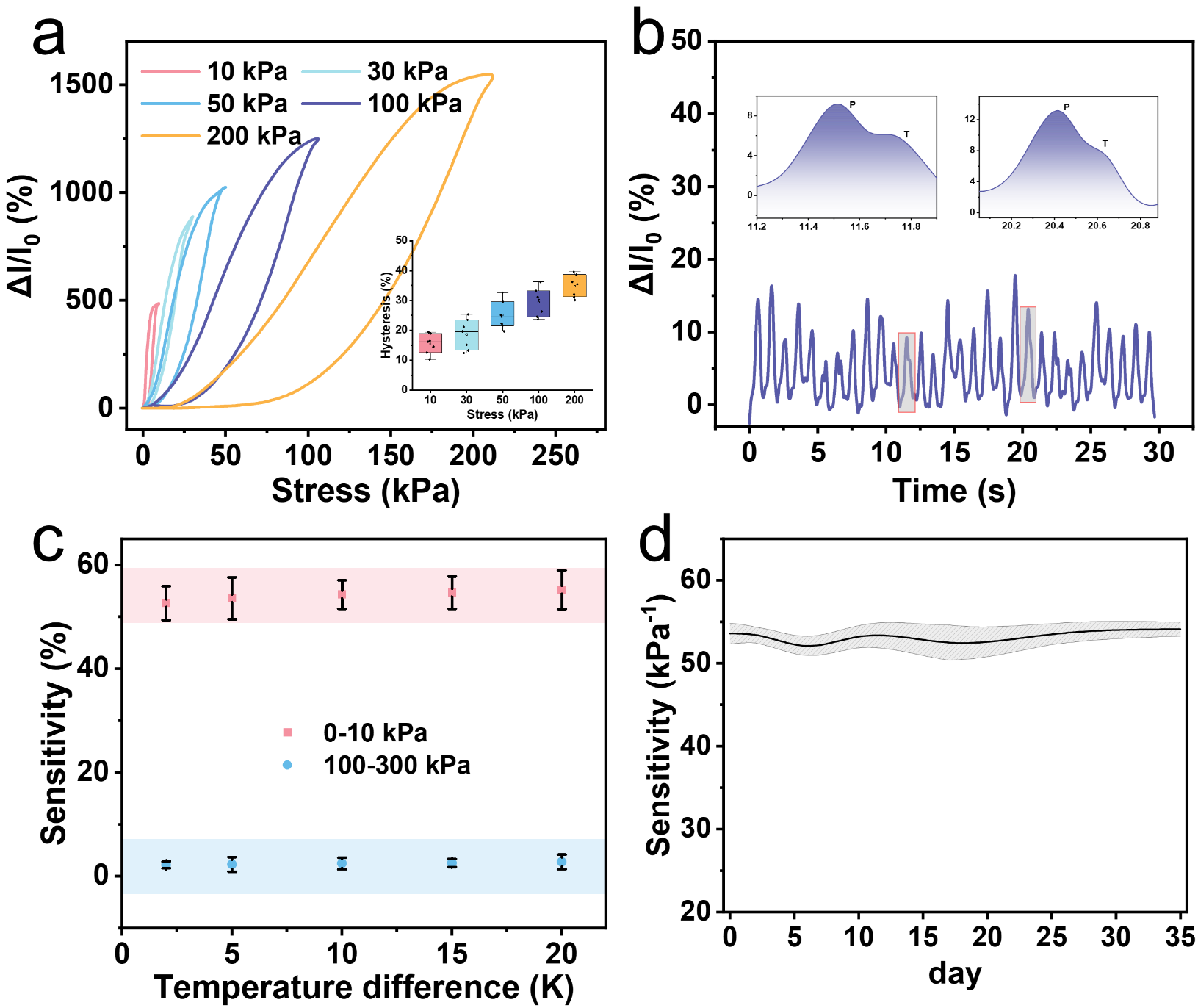


**Figure S15.** (a) The cycling curves of the GMTH at stress of 10-200 kPa. The inset is the calculated hysteresis statistics. (b) Monitoring of radial artery pulses using the GMTH. (c) The sensitivity of the GMTH under different temperature difference. (d) Long-term stability evaluation of the pressure sensing.


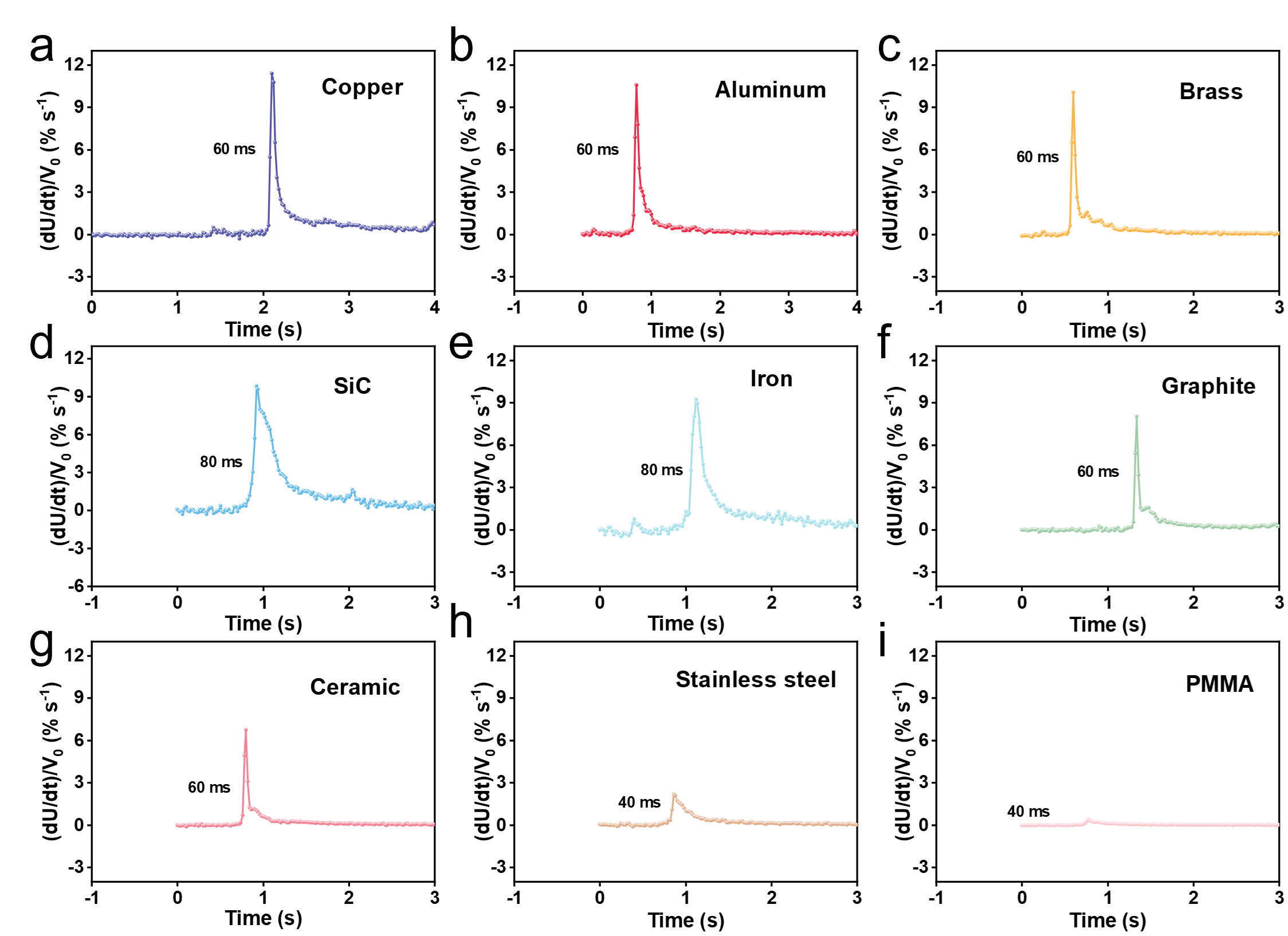


**Figure S16.** (a-i) Summary of the response time based on the differential voltage signals of 9 kinds of materials.


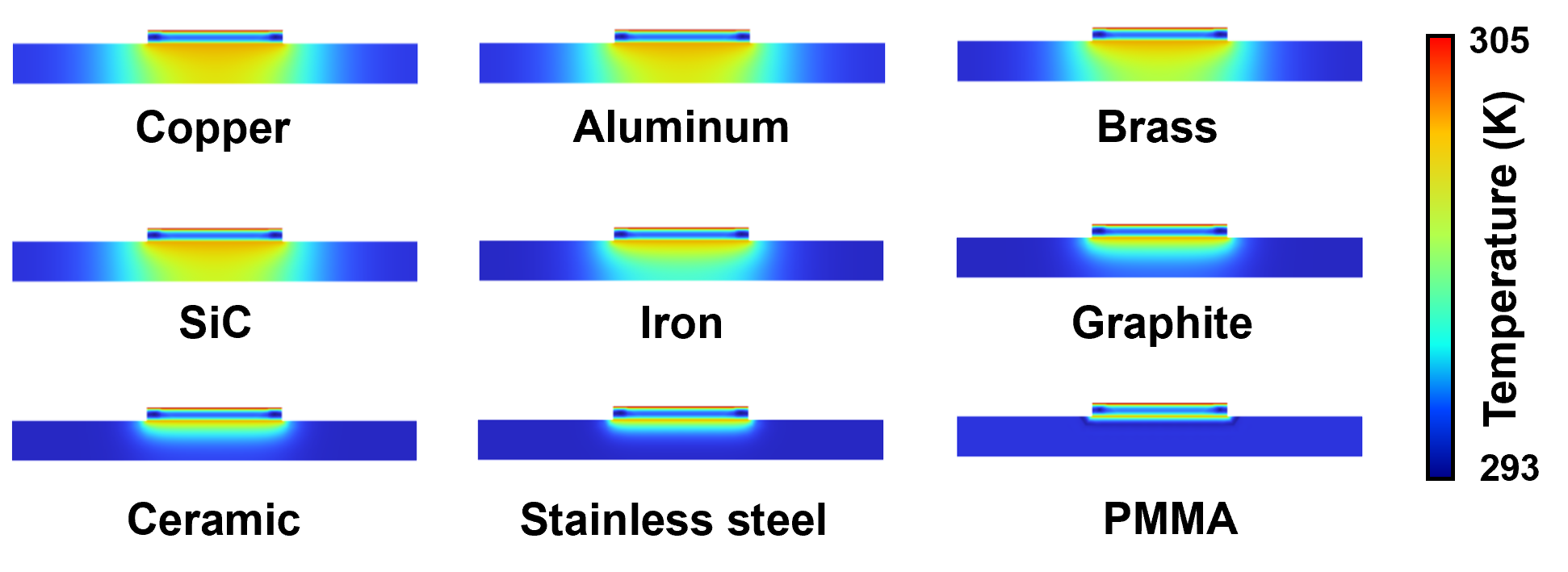


**Figure S17.** The temperature distributions and heat transfer capability of the objects are simulated when the receptor contacts various materials within 2 s.


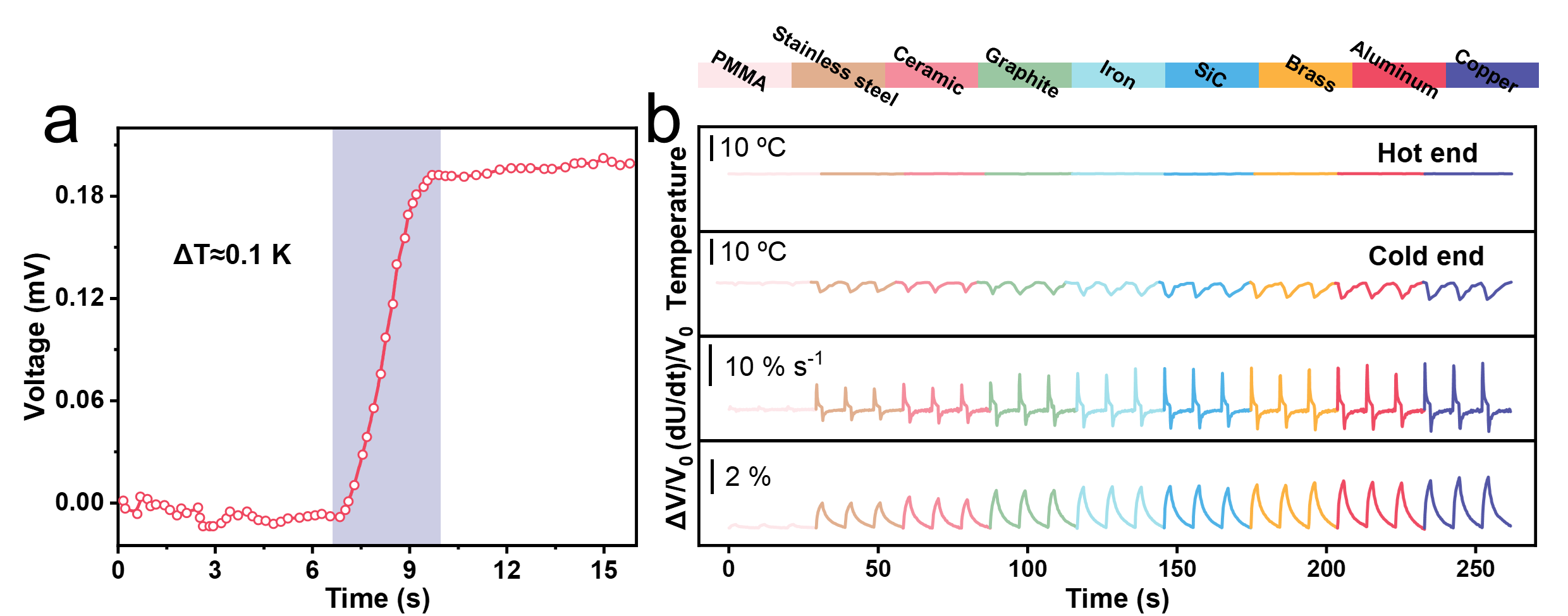


**Figure S18.** (a) Voltage outputs under a subtle temperature difference of 0.1 K. (b) Simultaneous recording temperature, differential voltage and thermovoltage signals for 9 kinds of materials.


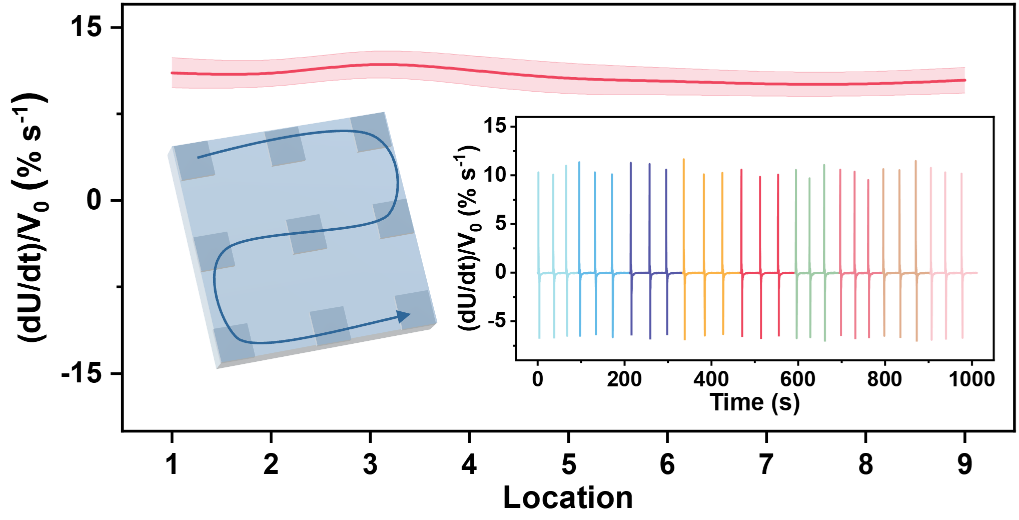


**Figure S19.** Responses of the receptor to contact materials at different locations.


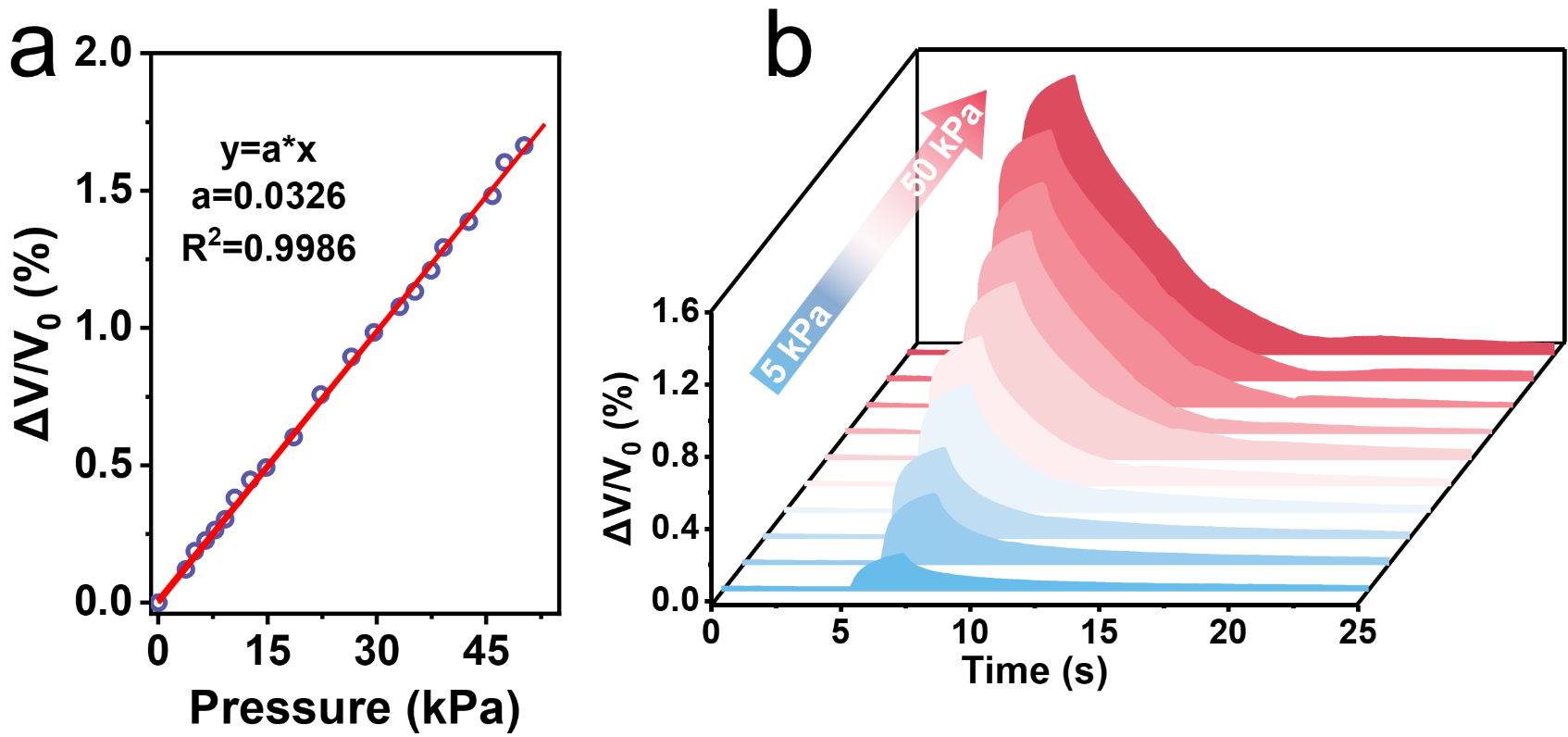


**Figure S20.** (a, b) Thermovoltage responses of the receptor to aluminum when contact pressure ranging from 5 to 50 kPa.


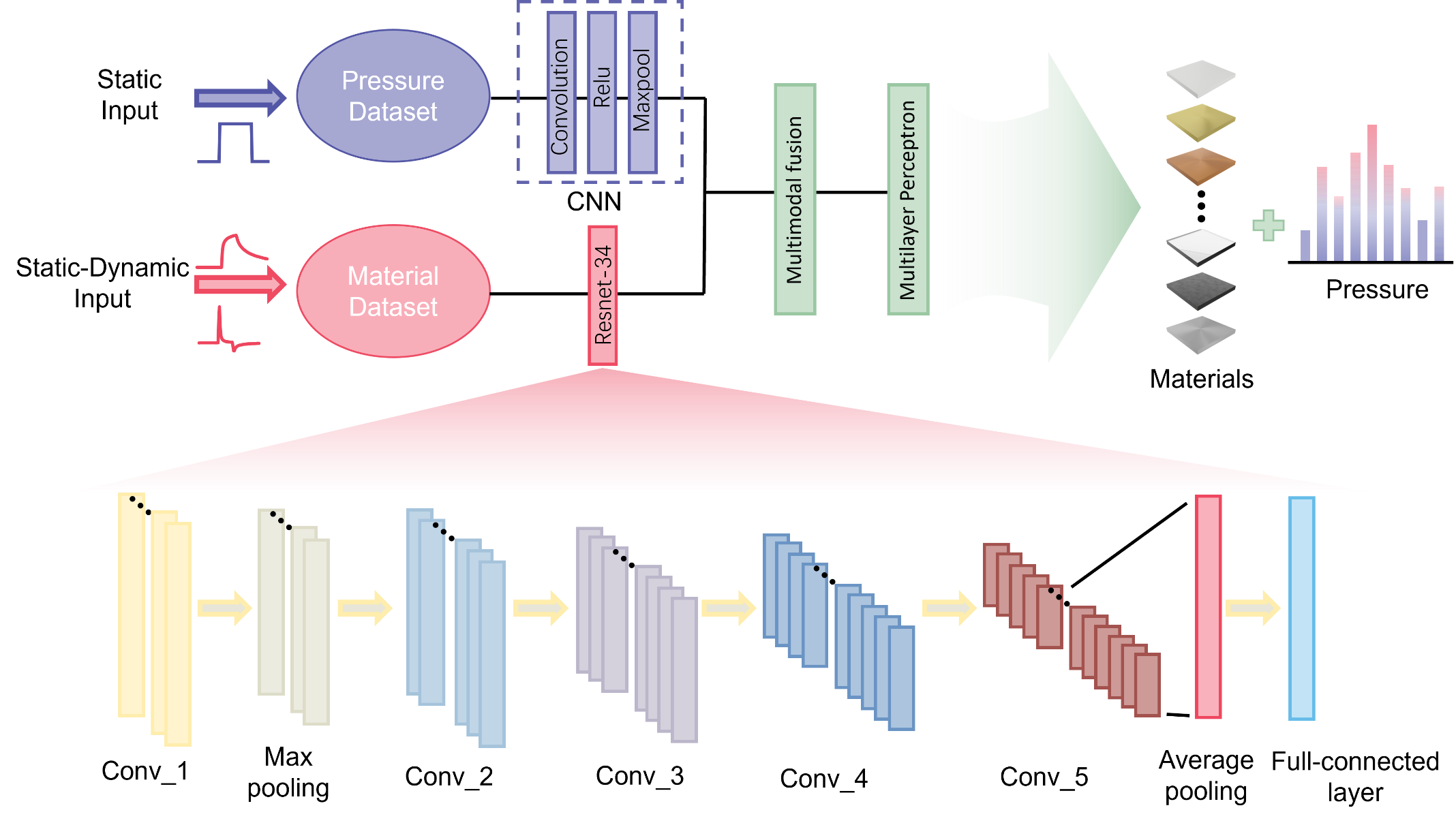


**Figure S21.** Multimodal deep learning algorithm architecture for self-supervised tactile perception.


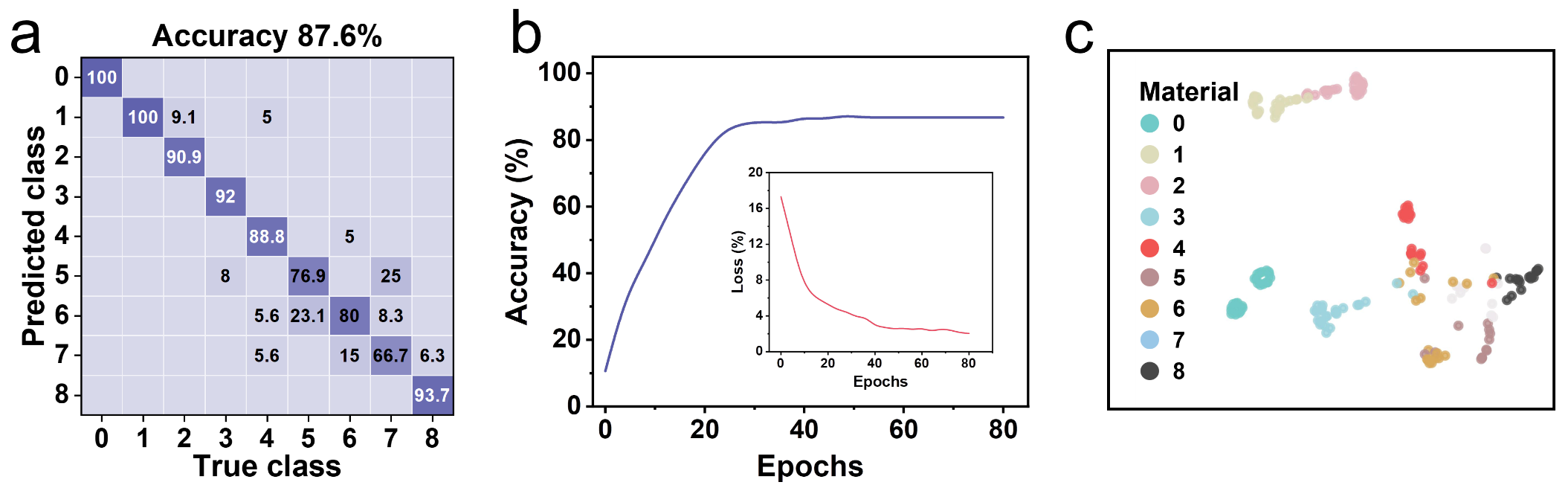


**Figure S22.** (a) Confusion matrix of recognizing materials using the voltage signals alone under a fixed contact pressure. Material types correspond to the numbers ranging from 0 to 8. (b) The corresponding accuracy and loss curves. (c) The distribution of datasets in a two-dimensional space.


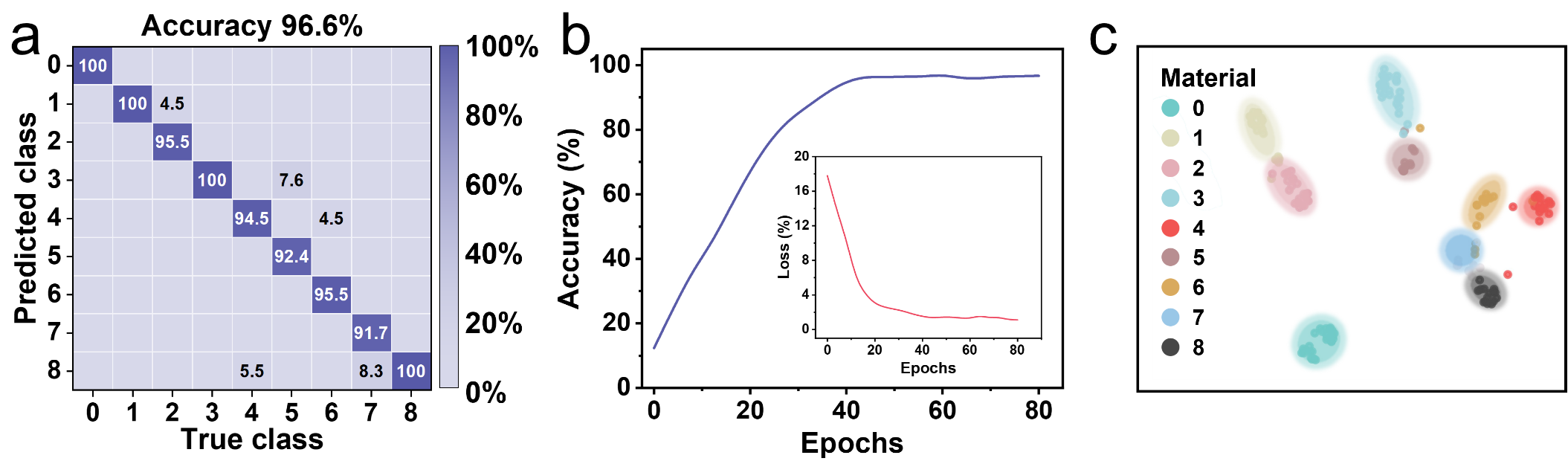


**Figure S23.** (a) Confusion matrix of recognizing materials using the voltage signals and the differential voltage signals under a fixed contact pressure. Material types correspond to the numbers ranging from 0 to 8. (b) The corresponding accuracy and loss curves. (c) The distribution of datasets in a two-dimensional space.


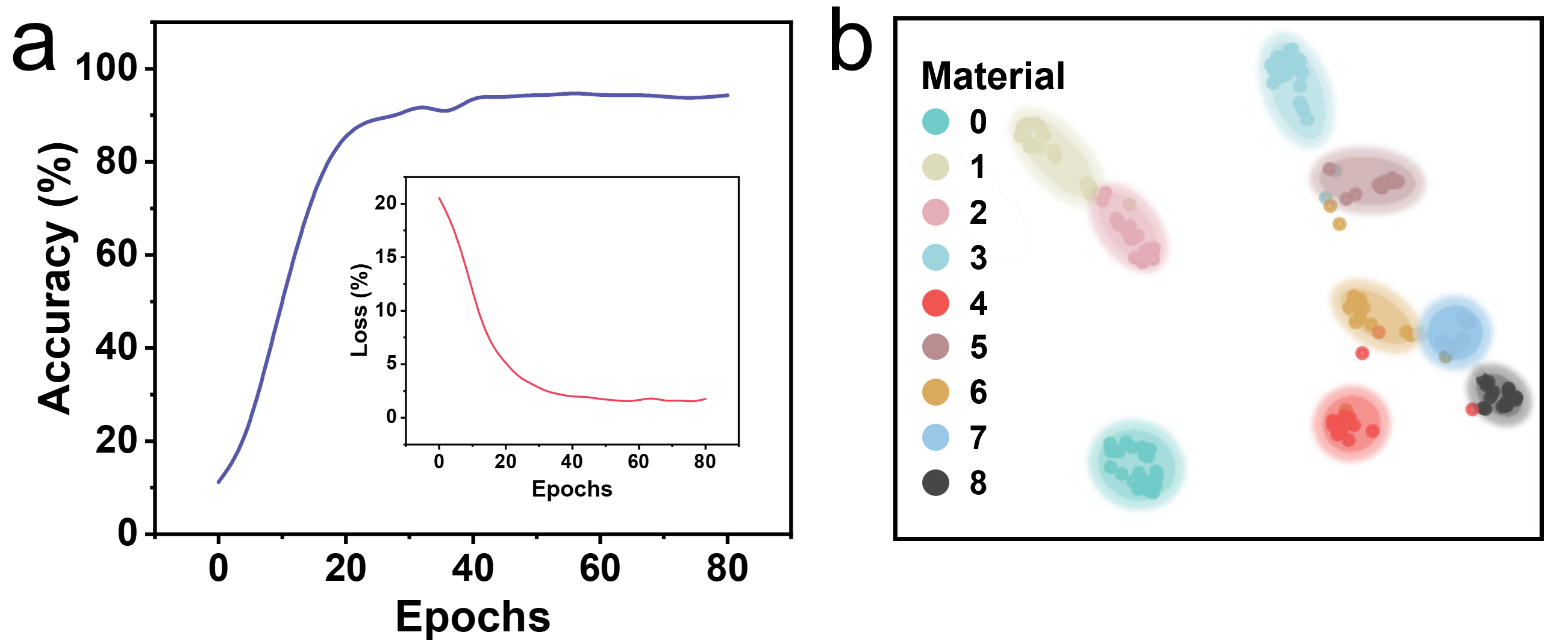


**Figure S24.** (a) The accuracy and loss curves in a self-supervised model. (b) The distribution of datasets in a two-dimensional space. Material types correspond to the numbers ranging from 0 to 8.

Table S1. Advancements in the mechanical, thermoelectric, and non-drying properties of thermogalvanic hydrogels.

| Stretchability  (%) | Stress  (MPa) | Se  (mV K^-1^) | Conductivity  (S m^-1^) | Non-drying  (>10 day) | Ref. |
| --- | --- | --- | --- | --- | --- |
| 611 | 10.98 | 1.89 | 2.74 | Yes | **This work** |
| 250 | 0.05 | 2.01 | 1.2 | Yes | [22] |
| 300 | 0.38 | 1.29 | 0.89 | Yes | [32] |
| 100 | 0.009 | 1.5 | 1.68 | No | [33] |
| 780 | 0.245 | 1.43 | 2.46 | No | [34] |
| 130 | 0.3 | 1.63 | 1.71 | No | [35] |
| 420 | 1.1 | 1.38 | 0.85 | Yes | [36] |
| 380 | 4.2 | 1.4 | 1.84 | No | [37] |
| 250 | 0.45 | 2.2 | 3.5 | Yes | [38] |
| 350 | 8.2 | 2.17 | 4.56 | No | [39] |

Table S2. A comparison of the GMTH with other representative reported hydrogels in terms of sensitivity, response time, and sensing range.

| Sensitivity (kPa^-1^) | | Response time (ms) | Sensing range (kPa) | Ref. |
| --- | --- | --- | --- | --- |
| 53.6 | | 110 | 500 | **This work** |
| 4.92 | 230 | | 20 | [3] |
| 0.012 | 120 | | 300 | [40] |
| 1.34 | 40 | | 180 | [41] |
| 7.36 | 260 | | 25 | [42] |
| 1.42 | 80 | | 36.75 | [43] |
| 0.053 | 20 | | 300 | [44] |
| 0.2 | 562 | | 10 | [45] |
| 5.92 | 50 | | 8.5 | [46] |
| 9.43 | 150 | | 60 | [47] |

**Supplemental references:**

[1] J. Duan, G. Feng, B. Yu, J. Li, M. Chen, P. Yang, J. Feng, K. Liu, J. Zhou, *Nat. Commun.* **2018**, *9*, 5146.

[2] B. Yu, J. Duan, H. Cong, W. Xie, R. Liu, X. Zhuang, H. Wang, B. Qi, M. Xu, Z. L. Wang, J. Zhou, *Science* **2020**, *370*, 342.

[3] G. Li, S. Liu, L. Wang, R. Zhu, *Sci. Robot.* **2020**, *5*, eabc8134.

[4] W. Yang, M. Xie, X. Zhang, X. Sun, C. Zhou, Y. Chang, H. Zhang, X. Duan, *ACS Appl. Mater. Interfaces* **2021**, *13*, 55756.

[5] H.-N. Ho, L. A. Jones, *J. Biomech. Eng.* **2008**, *130*, 021005.
